# Proximity-induced surface superconductivity in Dirac semimetal Cd_3_As_2_

**DOI:** 10.1038/s41467-019-10233-w

**Published:** 2019-05-17

**Authors:** Ce Huang, Benjamin T. Zhou, Huiqin Zhang, Bingjia Yang, Ran Liu, Hanwen Wang, Yimin Wan, Ke Huang, Zhiming Liao, Enze Zhang, Shanshan Liu, Qingsong Deng, Yanhui Chen, Xiaodong Han, Jin Zou, Xi Lin, Zheng Han, Yihua Wang, Kam Tuen Law, Faxian Xiu

**Affiliations:** 1 State Key Laboratory of Surface Physics and Department of Physics, Fudan University, Shanghai, 200433 China; 2 Institute for Nanoelectronic Devices and Quantum Computing, Fudan University, Shanghai, 200433 China; 3 Department of Physics, The Hong Kong University of Science and Technology, Clear Water Bay, Hong Kong, China; 4 Shenyang National Laboratory for Materials Science, Institute of Metal Research, Chinese Academy of Sciences, Shenyang, 110016 China; 5 International Center for Quantum Materials, Peking University, Beijing, 100871 China; 6 Materials Engineering, The University of Queensland, Brisbane, QLD 4072 Australia; 7 Beijing Key Laboratory of Microstructure and Property of Advanced Materials, Institute of Microstructure and Property of Advanced Materials, University of Technology, 100124 Beijing, China; 8 Centre for Microscopy and Microanalysis, The University of Queensland, Brisbane, QLD 4072 Australia; 9 Collaborative Innovation Center of Advanced Microstructures, Nanjing, 210093 China

**Keywords:** Superconducting properties and materials, Topological insulators, Superconducting devices

## Abstract

Cd_3_As_2_ is a three-dimensional Dirac semimetal with separated Dirac points in momentum space. In spite of extensive transport and spectroscopic studies on its exotic properties, the evidence of superconductivity in its surface states remains elusive. Here, we report the observation of proximity-induced surface superconductivity in Nb/Cd_3_As_2_ hybrid structures. Our four-terminal transport measurement identifies a pronounced proximity-induced pairing gap (gap size comparable to Nb) on the surfaces, which exhibits a flat conductance plateau in differential conductance spectra, consistent with our theoretical simulations. The surface supercurrent from Nb/Cd_3_As_2_/Nb junctions is also achieved with a Fraunhofer/SQUID-like pattern under out-of-plane/in-plane magnetic fields, respectively. The resultant mapping shows a predominant distribution on the top and bottom surfaces as the bulk carriers are depleted, which can be regarded as a higher dimensional analog of edge supercurrent in two-dimensional quantum spin Hall insulators. Our study provides the evidence of surface superconductivity in Dirac semimetals.

## Abstract (web summary)

Surface superconductivity in a Dirac semimetal remains rarely studied. Here, Huang and Zhou et al. report the evidence of proximity-induced surface superconductivity in a superconductor/Dirac semimetal hybrid system Nb/Cd_3_As_2_.

## Introduction

The study of topological phases has been one of the central topics in condensed matter physics in the past decade^1,2^. Among the rich class of topological phases, topological Weyl and Dirac semimetals, which are characterized by discrete gapless nodes in the bulk spectra, have attracted wide attention^3–5^. Referred to as Weyl or Dirac points, these gapless nodes in the bulk can be connected by open strings formed by topologically protected surface states on the boundaries, called Fermi-arc states^6^. Due to their anomalous electromagnetic responses^7–14^, as well as the interesting interplay between bulk and surface Fermi arcs^15,16^, topological semimetals have been studied intensively in recent years^5^. In parallel to the rapid exploration of Dirac properties in topological semimetals, the study of their superconducting states has become an important topic aiming for possible unconventional superconductivity^17–20^. It was predicted that Fermi-arc states in a Dirac semimetal such as Cd_3_As_2_ could be used to realize Majorana fermions^20,21^. This is due to the fact that the Fermi-arc states in Cd_3_As_2_ stem from helical edge states of quantum spin Hall insulators embedded in the time-reversal invariant semimetal^22^. By proximitizing the Fermi-arc states with *s*-wave superconductors, Majorana flat band can be possibly created at the interface of a π-Josephson junction^20,23^. While the signature of superconductivity has been found in Cd_3_As_2_^24–26^, the role of Fermi-arc states in these pairing phases remains unclear.

Despite the appealing proposals for creating Majorana fermions, the proposed scheme relies on the assumption^20,21^ that the Fermi arcs can acquire hard superconducting gaps from the parent superconductor. In topological insulator/superconductor (TI/SC) junctions, it is generally believed that the induced superconducting gap in the surface states is small due to high interfacial barriers and Fermi surface mismatch^27–29^. In a Dirac semimetal Cd_3_As_2_, the unavoidable coexistence of low-energy bulk excitations and Fermi-arc states^5^ causes further complications to distinguish the roles of bulk states and surface states in the superconducting state. Therefore, a prerequisite experimental evidence of strong superconducting pairing on the surface is clearly desirable. In particular, due to the highly non-uniform spatial distribution of surface states in Cd_3_As_2_, the superconducting proximity on the surface and bulk may exhibit distinctive anisotropic signatures.

Here, we report the observation of strong proximity-induced superconductivity on the surfaces of Cd_3_As_2_ based on two superconducting characteristics: the proximity-induced superconducting gap and the spatial distribution of supercurrents on surfaces. Using four-terminal transport measurements on Nb/Cd_3_As_2_ hybrid structures, we observe a pronounced proximity-induced gap (Δ_s_) on the surfaces, with its size comparable to the parent superconducting gap (Δ_Nb_) of Nb. The sizable surface proximity gap manifests itself as a flat conductance plateau in differential conductance (d*I*/d*V*) spectra. In contrast, the proximity gap in bulk states is a few times smaller (Δ_b_ ~ 0.14Δ_s_) and featured by a zero-bias broad peak (ZBBP) in the d*I*/d*V* spectra. The relation between the observed conductance plateau/ZBBP and proximity-induced gaps in surface/bulk states are further confirmed by our theoretical calculations with a qualitatively good agreement. Upon changing surface-bulk contributions in Cd_3_As_2_ samples with different thicknesses, the conductance plateau/ZBBP exhibit evolutionary behaviors consistent with their surface/bulk origins. Furthermore, using superconducting quantum interference (SQI) measurements on Nb/Cd_3_As_2_/Nb Josephson junctions (JJs), we observe a SQUID pattern in the surface-state-dominated Cd_3_As_2_ JJs, which indicates that the supercurrent density is predominantly on top and bottom surfaces.

## Results

### Proximity-induced superconductivity in Nb/Cd_3_As_2_

**Fig. 1:**
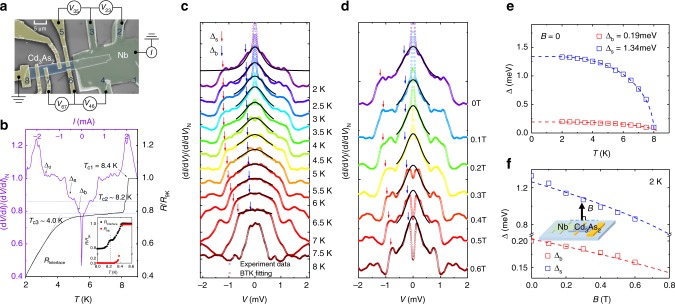
Proximity-induced superconductivity and differential conductance spectra in Nb/Cd_3_As_2_ hybrid structure. **a** False-color scanning electron microscopy image of the device with measurement configurations. Nb is deposited on top of Cd_3_As_2_ nanoplate. The thickness of Nb and Cd_3_As_2_ nanoplate is ~250 and ~200 nm, respectively. A four-terminal measurement across the interface was performed. A constant current (*I*) was applied through electrode 1 and 8 and *V*_ab_ is the voltage drop between electrode *a* and *b*. The voltage drop at different electrodes represents the resistance of that region. Scale bar, 5 μm. **b** The relationship of differential resistance spectra at 2 K (left with purple color) and the normalized *R–T* curve (right with black color) for interface resistance between electrodes 3 and 5. Blue dashed lines display different regions corresponding to each transition in the *R–T* curve. **c** Zero-field temperature-dependent d*I*/d*V* of *R*_Interface_, normalized by the normal-state conductance at 9 K. The curves are vertically shifted for clarity. Obvious features are observed as bias-independent conductance plateau (red arrows) (Δ_s_) and broad peak (blue arrows) (Δ_b_) from proximity-induced superconductivity in Cd_3_As_2_. Best fit of d*I*/d*V* to the BTK theory in ZBBP region is displayed. The colorized circle dots plot the experimental data while the black lines are fitting curves. **d** Magnetic field-dependent d*I*/d*V* of *R*_Interface_ at 2 K, normalized by the normal-state conductance at 9 K. Black lines are the BTK fitting of the broad peak. **e** Temperature dependence of Δ_d_ and Δ_s_. Dashed lines are the BCS fits. **f** Magnetic field dependence of Δ_d_ and Δ_s_. Dashed lines are the BCS fits. Inset displays a schematic drawing with the magnetic field direction perpendicular to the Nb/Cd_3_As_2_ plane

The proximity-induced superconductivity in Nb/Cd_3_As_2_ hybrid structure is displayed in Fig. 1a (device #01). The 250 nm-thick Nb film, colored in green in the false-color scanning electron microscopy (SEM) image, is deposited on top of a ~200 nm-thick Cd_3_As_2_ nanoplate (blue color). A four-terminal measurement across the interface was performed to detect the interface resistance, denoted as *R*_Interface_ (see Supplementary Note 1, Supplementary Fig. 1 for sample characterizations and Supplementary Note 2, Supplementary Figs. 2 and 3 for measurement details).

Temperature-dependent resistance (*R*–*T*) curves at *T* ≥ 2.0 K across the junction are shown in Fig. 1b (black curve) with three drops at *T*_c1_ = 8.4 K, *T*_c2_ ~ 8.2 K, and *T*_c3_ ~ 4.0 K corresponding to Nb superconducting for *T*_c1_ and proximity-induced superconductivity for *T*_c2_ and *T*_c3_, respectively, since Nb has been in zero-resistance state at 8.3 K (Fig. 1b inset, also see details in Supplementary Note 3 and Supplementary Fig. 4). Then, we carry out the four-terminal d*I*/d*V* measurements to show the induced superconductivity in Cd_3_As_2_ in Fig. 1b–d. A bias-independent conductance plateau (BICP) appears in the vicinity of ±1.20 mV (Δ_s_) with a ZBBP around ±0.19 mV (Δ_b_) and an above-gap dip at high bias (Δ_d_). For temperatures lower than 4 K, a very sharp zero-bias conductance peak (ZBCP) emerges which is superimposed on the broad peak (Fig. 1c). Control experiments exclude the effect from Nb or Cd_3_As_2_ (Supplementary Note 3 and Supplementary Figs. 5 and 6). Evidently, the ZBBP and ZBCP correspond to the second transition (*T*_c2_) and third transition (*T*_c3_) in the *R*–*T* curve while the first transition (*T*_c1_) mostly corresponds to the conductance plateau with a small resistance drop by the above-gap dip in Fig. 1b. The observation of the superconducting proximity effect and conductance plateau suggests that the interface is of high transparency. Fits to the broad peaks in black lines by standard Blonder–Tinkham–Klapwijk (BTK) theory^30^ in Fig. 1c, d confirm the proximity-induced superconductivity in Cd_3_As_2_. The BTK fittings yield important parameters of Δ_b_ = 0.19 meV and Z = 0 (Supplementary Note 4 and Supplementary Figs. 7 and 8). Obviously, the amplitudes of BICP, ZBBP and ZBCP decrease with increasing temperature and perpendicular magnetic field. Figure 1e, f, respectively, show temperature- and magnetic field-dependent gaps which are fitted to the Bardeen–Cooper–Schrieffer (BCS) theory (Supplementary Note 5). Δ_s_ is extracted to be 1.34 meV at 0 T, comparable to the superconducting gap of Nb estimated from the BCS theory^31^ Δ = 1.76*k*_B_*T*_c_ ~ 1.30 meV, which further verifies the proximity effect in the Nb/Cd_3_As_2_ system and *s*-wave pairing in Cd_3_As_2_.

The above-gap dip Δ_d_ and ZBCP are, respectively, attributed to depairing effect near the interface and the proximity-induced superconductivity in another band of Cd_3_As_2_ such as the band projected onto [112] direction (Supplementary Note 6 and 7 and Supplementary Figs. 9–11).

**Fig. 2:**
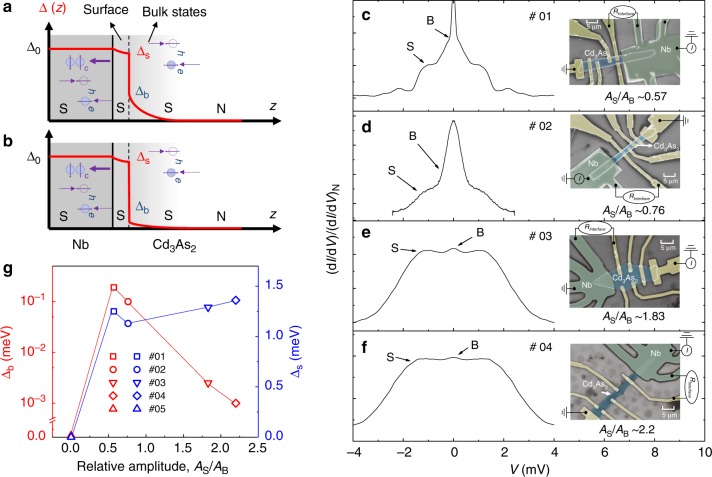
d*I*/d*V* curves in Cd_3_As_2_ with different bulk/surface channels domination by controlling Cd_3_As_2_ thickness. **a**, **b** A schematic drawing of the hybrid structure in two regimes from top to bottom: bulk and surface channels coexisting with the proximity-induced superconductivity in both bulk and surface channels; surface-channel-dominated Cd_3_As_2_ with fast decay superconducting order parameters in the bulk regime. Andreev reflection takes place in the whole proximity region with lower energy particles reflected mainly from the superconducting bulk channels of Cd_3_As_2_, and the higher energy ones mainly from the proximity-induced surface superconductivity. **c**–**f** Normalized difference conductance spectra of *R*_Interface_ in device #01, #02, #03, #04 at 2 K, respectively. Right insets display false-color SEM images of the devices. Scale bars, 5 μm. From (**c**) to (**f**), the Cd_3_As_2_ becomes thinner and surface states gradually dominate the transport. We use the relative amplitude of the surface oscillations compared with the bulk oscillation *A*_S_/*A*_B_ to estimate the surface/bulk channels (S/B) domination. The d*I*/d*V* shape evolves from BICP and broad peak in (**c**) and (**d**) to single plateau in (**e**) and (**f**). **g** The summary of Δ_s_ and Δ_b_ with a relative amplitude of the surface oscillations compared with the bulk oscillations, for magnetic field perpendicular to the surface at 2 K. Red and blue spots and lines are for Δ_s_ and Δ_b_, respectively

Next, we probe the relation between BICP, ZBBP and proximity-induced superconductivity. For voltage bias *V* lower than the Nb superconducting gap, the transport process is dominated by Andreev reflection (AR), in which an incoming electron from the Dirac semimetal is converted to a reflected hole at the Nb/Cd_3_As_2_ interface. Thus, the presence of both ZBBP and BICP for sub-gap voltage bias suggests two types of Andreev reflection channels in Cd_3_As_2_: one exists only within a narrow energy window given by the width of the broad peak, while the other has a much wider energy range measured by the width of the conductance plateau. Since the energy window for Andreev reflections is directly related to the interfacial coupling between the channels of Cd_3_As_2_ and Nb, the contrasting features of the broad peak and plateau also suggest very different proximity effects in two types of channels. By placing Cd_3_As_2_ next to the superconducting Nb, the superconducting proximity effect is expected to occur in both the bulk and the surface region, as shown in Fig. 2a. Importantly, since the wave functions of the surface states are predominantly localized near the interface, the surface states can strongly couple to Nb than the bulk. As a result, it is reasonable to expect that the proximity effect in the surface states can be much stronger than that in the bulk states. Besides, when the Fermi level is close to the Dirac points, AR amplitude for bulk-state channels is expected to be small and the Cooper pair wave function would decay rapidly in the bulk region as shown in Fig. 2b.

To further distinguish the proximity effect on surface and bulk channels, we acquired the continually evoluting features of BICP and ZBBP by tuning the thickness to change the specific surface area with bulk/surface states proportion in Cd_3_As_2_ (Fig. 2c–f and Supplementary Note 8, Supplementary Figs. 12–15). We measured d*I*/d*V* spectra of Cd_3_As_2_ hybrid structure with different thickness at 2 K as shown in Fig. 2c–f. Devices # 01 and 02 with a thickness of ~200 nm both show BICP and ZBBP while the plateau remains pronounced while the ZBCP becomes strongly suppressed and hardly observable in thinner samples #03 and #04 (<150 nm), as shown in Fig. 2e, f, indicating that the surface channels dominate the proximity effect in thin samples. Thus, the channels responsible for the plateau are indeed from Cd_3_As_2_ surface states while the broad peak results from low-lying bulk channels in Cd_3_As_2_.

Next, we discuss the possible origin of surface superconducting channels. In device #01, the bulk states and Fermi-arc surface states can coexist (Table 1). As reported previously^16,32^, the Fermi surface property in Cd_3_As_2_ is thickness-dependent. In particular, we note that in samples with thickness >150 nm, the Fermi level is generically well above the Dirac points. Thus, both bulk states and Fermi-arc states contribute to electronic transport, while in samples thinner than 150 nm, the Fermi level is closer to the Dirac cone and Fermi-arc states dominate. Since both the electronic transport and proximity effects are closely related to the Fermi surface property, we anticipate that the surface channels possibly correspond to Fermi-arc surface states.

**Table 1:** Estimated band and superconducting proximity effect parameters in Cd_3_As_2_ at 2 K

Device	*t* (nm)	*F*_B_ (T)	*F*_S_ (T)	*A*_S_/*A*_B_	*ξ*_N_ (nm)	Δ_b_ (meV)	Δ_s_ (meV)
#01	~200	20.5	30.8	0.57	424	0.19	1.25
#02	~200	~7.9	23.6	0.76	263	0.10	1.13
#03	<150	~4.7	15.8	1.83	270	~2.5 × 10^−3^	1.29
#04	<150	~3.0	12.3	2.2	228	~1.0 × 10^−3^	1.36
#05	>300	33.0	/	~0	417	0	0
Moll et al.^32^	/	36.5	61.5	/	/	/	/

The bulk and surface frequency *F*_B_ and *F*_S_ can be extracted from the SdH oscillations. The superconducting proximity-induced surface states gap Δ_s_ and bulk states gap Δ_b_ can be extracted from the BTK fitting of differential conductance spectra

Both devices # 01 and 02 have shown the bulk states and Fermi arcs in transport from SdH oscillation measurements. Here, we use the relative amplitude of the surface oscillations compared with the bulk oscillation *A*_S_/*A*_B_ to estimate the surface/bulk channels (S/B) domination. In contrast, thinner samples (# 03 and 04) exhibit low bulk domination. Based on the analysis of the SdH oscillations (Supplementary Note 9, Supplementary Fig. 18 and Supplementary Table 1), the Fermi surface property is confirmed to be dominated by the Fermi-arc states. Therefore, through the different weight of surface-bulk conduction, we experimentally attribute the flat conductance plateau to the superconducting proximity effect possibly in the Fermi-arc states and the broad peak to the relatively weak proximity effect in the bulk states. Besides, the surface and bulk contribution to Andreev reflection is also consistent with the conductance enhancement (Supplementary Note 10 for details).

In the thick limit of Cd_3_As_2_ where bulk states dominate, weak Andreev reflection occurs with strong tunneling behavior as a result of insufficient electrons from the bulk states to participate in AR^30^ (Supplementary Note 8, Supplementary Figs. 12–14, device #05, >300 nm thick). At the interface, the AR electron density is proportional to $\frac{{{\mathrm{d}}I}}{{{\mathrm{d}}V}} \propto G\left( {E_{\mathrm{F}}} \right) \propto \frac{{4e^2}}{h}N\left( {E_{\mathrm{F}}} \right) \propto k_{\mathrm{F}}^2 + k_0^2$, where *k*_0_ is Fermi-arc length and can be estimated by^15^

1 $$F_{\mathrm{s}} = E_{\mathrm{F}}k_0/(e\pi v_{\mathrm{F}}),$$

where *F*_s_ is surface frequency. The Fermi velocity *v*_F_ = ℏ*k*_F_/m* can be extracted from the bulk SdH oscillations. In device #01, *k*_F_ ~ 0.0249 Å^−1^ and *k*_0_ ~ 0.8 nm^−1^ (ref. ^32^), and *k*_F_ = 0.032 Å^−1^ in device #05 with negligible *k*_0_, thus the AR electron density in device #01 is much higher than that in device #05. Furthermore, scattering effects between the bulk and surface states can be significant due to disorders, and the surface states are no longer well-localized on the surfaces^33^ which reduce the effective coupling between the surface and Nb. Besides, for high Fermi levels, there could possibly exist a significant Fermi surface mismatch between the superconductor and the Cd_3_As_2_, which could further suppress the interfacial coupling.

We now analyze the proximity-induced superconducting gaps in Cd_3_As_2_. Two superconducting gaps against the relative amplitude of *A*_S_/*A*_B_ are presented in Fig. 2g and Table 1. The wave functions of the surface states are localized at the surface, while these of the bulk states are predominantly in the bulk. Effectively, the surface states couple much more strongly to the superconductor than the bulk states. In thin Cd_3_As_2_, the transparent interface with enough electron states from surface channels ensures that the proximity effect arises. Hence, the bulk-state superconducting gap Δ_b_ drops fast while the surface superconducting gap Δ_s_ increases slightly with a larger surface domination. The penetration depth for each surface can be estimated with an assumption that the superconducting gap decreases following^34^

2 $${\mathrm{\Delta }}\left( z \right)\sim {\mathrm{\Delta exp}}\left( { - \frac{z}{{\xi _{\mathrm{N}}}}} \right),$$

where *ξ*_N_ is the superconducting coherence length in Cd_3_As_2_ (Table 1) and *z* is the distance in Cd_3_As_2_ from the interface. At the surface regime, ${\mathrm{\Delta }}_{{\mathrm{Nb}}} = {\mathrm{\Delta }}_{\mathrm{s}}{\mathrm{exp}}\left( { - \frac{{z_{\mathrm{s}}}}{{\xi _{\mathrm{N}}}}} \right)$, we can evaluate the surface penetration depth *z*_s_ in Cd_3_As_2_ of 10–50 nm, which is similar to the previous report for Fermi arcs in Cd_3_As_2_^16^ and agrees with other numerical simulations based on the low-energy model of Dirac semimetals^35^ as an evidence of possible Fermi-arc superconductivity.

### Theoretical calculations on Andreev reflection

**Fig. 3:**
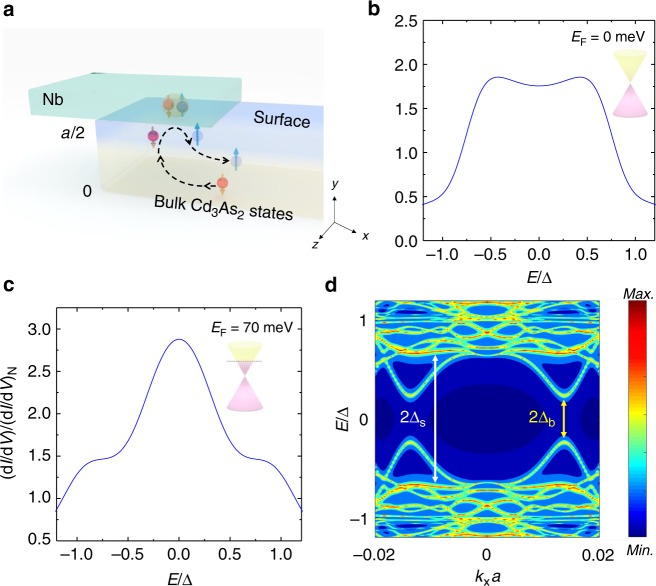
Numerical calculations of d*I*/d*V* and the superconducting proximity effect for the Nb/Cd_3_As_2_ hybrid structure. **a** A schematic sketch showing the Andreev reflections and the superconducting proximity effect in the Nb/Cd_3_As_2_ junction. When superconducting Nb layer is on the top of Cd_3_As_2_ with a thickness of *a*, proximity-induced cooper pairs exist in both bulk states and surface states. The red and blue spheres represent the electrons and holes with opposite spin directions. The dashed line displays the Andreev reflection process. **b**, **c** Numerical calculations of the normalized Andreev reflection amplitude in Nb/Cd_3_As_2_ junction: **b** Andreev reflection amplitude for Fermi level lying at the Dirac points (*E*_F_ = 0 meV). In this case, the bulk density of states is negligible and Fermi-arc states dominate the Andreev reflections. Clearly, only a flat conductance plateau is found in the d*I*/d*V* spectrum; **c** Andreev reflection amplitude for Fermi level lying high above the Dirac points (*E*_F_ = 70 meV), where the bulk density of states cannot be ignored. A broad zero-bias peak emerges on top of the flat plateau in the d*I*/d*V* spectrum. **d** Spectral density on the topmost surface of Cd_3_As_2_ for *E*_F_ = 70 meV, with the contribution of superconducting Nb integrated out and projected on the surface of Cd_3_As_2_. The color bar shows the local density of states on the top Cd_3_As_2_ surface on a logarithmic scale. In the color scale, red (blue) color indicates high (low) density. Evidently, two pairing gaps are induced on the surface. The larger proximity gap Δ_s_ originates from Fermi-arc states (with high local density on the surface). Notably, 2Δ_s_ corresponds to the width of the flat conductance plateau due to surface Fermi arcs in (**b**) and (**c**). In contrast, the smaller gap Δ_b_ originates from bulk states (with low density on the surface). Consistently, 2Δ_b_ roughly measures the width of the zero-bias peak due to bulk states in (**c**)

Our experimental observations above are further supported by theoretical calculations of the d*I*/d*V* spectra for our Nb/Cd_3_As_2_ hybrid structure. Using numerical Green’s function method based on a four-band tight-binding model of Cd_3_As_2_, we calculate the Andreev reflection amplitude of the Nb/Cd_3_As_2_ junction with the parent superconductor Nb modeled by a usual *s*-wave superconductor with the schematic set-up in Fig. 3a. Details of the Hamiltonian and the Green’s function are presented in the Method section, Supplementary Note 11, Supplementary Figs. 19 and 20 and Supplementary Table 2.

It is worth noting that due to the low-energy Dirac spectrum of Cd_3_As_2_, the bulk density of states is expected to increase monotonically as a function of *E*_F_ measured from the Dirac points located at (0, 0, ±*k*_0_). In contrast, Fermi-arc states with different energies are connected on the surface, thus the number of Fermi-arc channels on the junction interface is expected to depend weakly on the Fermi level. Therefore, tuning the chemical potential *E*_F_ of Cd_3_As_2_ in our model allows us to theoretically investigate the roles of surface states and bulk states in the Andreev reflection processes.

First, we consider the case that the location of chemical potential is at the Dirac points (*E*_F_ = 0 meV), where the bulk density of states is very low and the Andreev reflections driven by bulk channels are negligible. This simulates the scenario in thin samples (devices #03 and #04) where the Fermi surface property is dominated by Fermi-arc states. As shown in Fig. 3b, in this case only a flat conductance plateau is found in our simulations. Being consistent with our experimental observations, the signatures of ZBBP are hardly observable. Then, by tuning the Fermi level to *E*_F_ = 70 meV from the Dirac points, the bulk density of states gets enhanced and its contribution to Andreev reflections cannot be ignored. This simulates the case of relative thick samples (devices #01 and #02) where the bulk states and surface Fermi arcs have comparable weights in transport. In this scenario, a ZBBP emerges on top of the plateau in the differential conductance spectrum (Fig. 3c). Therefore, our theoretical calculations of the d*I*/d*V* spectrum suggest that the Fermi-arc channel can result in the plateau, while the ZBBP arises from Andreev reflections in the bulk-state channel. Here, we briefly note that without Fermi-arc states, a well-defined flat conductance plateau cannot be found in the d*I*/d*V* spectrum. This further indicates that the plateau most likely originates from Fermi-arc states. The detailed results are presented in Supplementary Note 11, Supplementary Fig. 21.

To understand the physical mechanisms for the formation of zero-bias peak and flat plateau in the d*I*/d*V* curve, it is worth noting that the energy windows for Andreev reflections driven by surface/bulk-state channels are measured by the widths of the plateau/zero-bias peak, respectively. This indicates that the superconducting proximity effect in the Fermi-arc states is much stronger than that in the bulk states. To demonstrate the proximity effects in Fermi-arc and bulk-state channels, we integrate out the superconducting Nb and include its contribution as a self-energy term on the topmost layer of Cd_3_As_2_ which interfaces with superconducting Nb. Considering the scenario where the bulk states have a non-negligible density of states, we use the same parameters in obtaining Fig. 3c to calculate the local spectral density on the topmost layer, as shown in Fig. 3d. The color bar indicates the local density of states on a logarithmic scale, with red (blue) colors indicating high (low) density. Clearly, due to the proximity-coupled *s*-wave superconductor, the topmost layer of Cd_3_As_2_ acquires two different spectral gaps, as indicated by white and yellow double arrows. Notably, the larger pairing gap is induced in states predominantly on the surface (higher local density of states on the surface), with twice of the gap size 2Δ_s_ corresponding to the width of the conductance plateau due to Fermi arcs, which is comparable to twice value of the parent superconducting gap. In contrast, the smaller induced gap is formed in states with lower density on the surface, and its size 2Δ_b_ matches the width of the zero-bias peak due to bulk states. This further confirms that the Fermi-arc states couple strongly to the superconductor, and thus acquire a sizable superconducting gap (~70% of parent gap Δ). In contrast, the states living in the bulk couple relatively weakly to the superconductor, which results in a smaller proximity gap. Therefore, the flat conductance plateau in the d*I*/d*V* curve indicates Fermi-arc superconductivity as a possible interpretation, while the zero-bias peak arises from relatively small proximity gap in the bulk states, which opens a narrower window near zero bias for Andreev reflections driven by bulk channels.

### Surface supercurrent in Nb/Cd_3_As_2_/Nb Josephson junctions

Having established the proximity-induced surface superconductivity in Nb/Cd_3_As_2_, we next build Nb/Cd_3_As_2_/Nb Josephson junctions to directly analyze the spatial distribution of the supercurrent. This is enabled by SQI measurements in different directions.

**Fig. 4:**
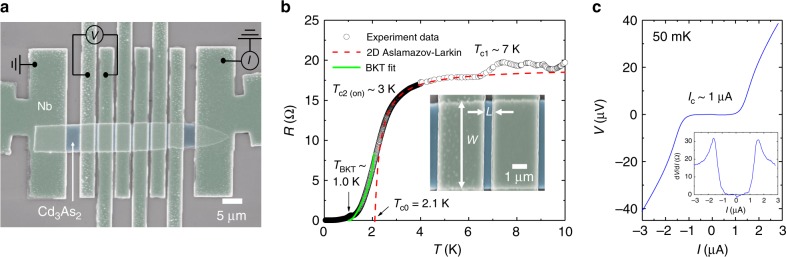
Characterization of Nb/Cd_3_As_2_/Nb Josephson junctions. **a** An SEM picture of a lateral Josephson junction based on a 120-nm-thick Cd_3_As_2_ nanoplate with 140-nm-thick Nb (device #06). Transport measurements on such a junction are performed in current-bias mode while measuring the voltage across the junction to investigate superconducting transport through the surface states. Scale bar, 5 μm. **b** Temperature dependence of Cd_3_As_2_ Josephson junction resistance for device #06 by a four-terminal method. Two transitions are identified: *T*_c1_ = 7 K is from superconducting Nb, *T*_c2(on)_ ~ 3 K is from the emergence of superconducting Cd_3_As_2_ and supercurrent is identified at *T*_c2(off)_ ~ 1 K. The red dashed line represents the superconducting amplitude fluctuation taking into account the 2D Aslamazov–Larkin model, which give the temperature, *T*_c0_ = 2.1 K, at which the finite amplitude of the order parameter develops. The green solid line represents the BKT transition using the Halperin–Nelson equation which gives a BKT transition temperature *T*_BKT_ = 1.0 K. Inset shows that the junction has a width of *W* = 7 μm and length of *L* = 500 nm. **c** *I–V* characteristics for Josephson junction in the superconducting states with a critical current of *I*_c_ ~ 1 μA under zero magnetic field at 50 mK. Inset: d*V*/d*I* characteristics indicates zero resistance below critical current, same as the *I*−*V* curve

Figure 4a schematically shows the lateral Cd_3_As_2_ Josephson junctions with closely spaced superconducting Nb electrodes on the top surface. We choose 120-nm-thick Cd_3_As_2_ with 140-nm-thick Nb electrodes to study the surface dominated supercurrent. The inset of Fig. 4b shows an SEM image of device #06. The length and width of the superconducting channel are *L* = 500 nm and *W* = 7 μm, respectively. Figure 4b shows the *R*–*T* curve of the junction with two transitions *T*_c1_ and *T*_c2_ at zero magnetic field. *T*_c1_ ~ 7 K originates from the Nb superconducting transition while *T*_c2(on)_ ~ 3 K comes from the superconducting proximity effect. The resistance continues to decrease as the junction cools down and reaches the zero-resistance state below *T*_c2(off)_ ~ 1 K. The tail of the resistance drop can be explained in terms of the BKT transition^36^ as shown in green line by Halperin–Nelson equation^37^

3 $$R = R_0{\mathrm{exp}}\left\{ { - 2b\left( {\frac{{T_{{\mathrm{c}}0} - T}}{{T - T_{{\mathrm{BKT}}}}}} \right)^{1/2}} \right\},$$

where *R*_0_ and *b* are material parameters, which realizes a zero-ohmic-resistance state driven by the binding of vortex-antivortex pairs at the BKT transition temperature *T*_BKT_ = 1.0 K. On the other hand, junction resistance decreases at the temperature above *T*_c2_, leading to a broadened SC onset, which can be well reproduced by the Aslamazov–Larkin^37^ fit in red dashed line for the two-dimensional (2D) fluctuation conductivity. These behaviors are consistent with expectations for 2D superconductivity as an evidence for the 2D surface superconductivity in Cd_3_As_2_ JJ since the thickness of Cd_3_As_2_ is far away from the 2D condition. Figure 4c and inset display the current-voltage (*I*−*V*) characteristics and differential resistance (d*V*/d*I*) of the junction measured at 50 mK, respectively. From the slope of the *I*−*V* curve, the normal-state resistance *R*_n_ ~ 17 Ω is extracted. In the regime |*I*| < 1 μA, the voltage across the junction and the d*V*/d*I* are zero, indicating a robust Josephson effect. The *I*_c_*R*_*n*_ product gives a characteristic voltage of ~17 μV which is lower than the transition temperature of 1 K (with superconducting gap Δ = 1.76*k*_B_*T*_c_ ~ 150 μV). It indicates that the junction is in the long junction limit^38^, where the superconducting coherence length *ξ*_N_ is smaller than the effective width between two Nb electrodes, i.e., *ξ*_N_ < *L*_eff_ (Supplementary Note 12).

**Fig. 5:**
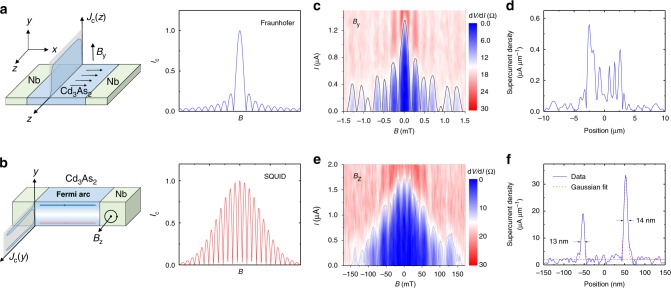
Evolution of surface superconductivity in the Josephson junction. **a** Left: a schematic picture of a lateral Josephson junction with the magnetic field along the out-of-plane direction. The Cd_3_As_2_ is filled with charge carriers and supercurrent can flow uniformly across the junction along the *z*-axis, corresponding to a flat supercurrent density *J*_c_(*z*) and the corresponding superconducting quantum interference has a Fraunhofer-like shape with a central lobe of width 2Φ_0_ and side lobes of width Φ_0_ (right part). **b** Left: schematic picture of Josephson junction with the magnetic field along in-plane direction. The top and bottom surface is filled with surface channels and supercurrent flows mostly on the two surfaces of Cd_3_As_2_, corresponding to a localized current density profile at the edges (upon *y*-axis). In this regime, the interference results in a sinusoidal double-slit pattern, with an overall decay in *B* that is determined by the width of the edge channels. **c** A map of the differential resistance across the junction at 35 mK with black guide line, showing the single-slit interference characteristics of a uniform supercurrent density. **d** The supercurrent distribution along *z*-axis, which was calculated as the inverse Fourier transform of the data in (**c**), consistent with trivial charge transport along the *z*-axis. **e** The map of the differential resistance with *B*_z_, showing a SQUID-like pattern. **f** The supercurrent distribution along *y*-axis. The supercurrent density is clearly dominated by the contribution from the two side edges along *y*-axis, indicating the surface superconductivity. The thickness of surface channels can be estimated using a Gaussian line shape by the red dashed line

The spatial distribution of supercurrent in a Josephson junction can be extracted by SQI measurements, where a magnetic field *B* perpendicular to the junction induces oscillations in the amplitude of the superconducting current. Measuring the dependence of the critical current $I_{\mathrm{c}}^{{\mathrm{max}}}$ on *B* provides a convenient way to extract the distribution of supercurrent which is widely used to probe edge-mode superconductivity in quantum spin Hall insulator and quantum Hall systems^39–41^. The particular shape of the critical current interference pattern depends on the phase-sensitive summation of the supercurrents traversing the junction. In the case of a symmetric supercurrent distribution, this integral takes the simple form:

4 $$I_{\mathrm{c}}^{{\mathrm{max}}}\left( B \right) = \left| {\mathop {\smallint }\limits_{ - \infty }^\infty J_{\mathrm{c}}\left( x \right){\mathrm{cos}}\left( {\frac{{2\pi L_{{\mathrm{eff}}}B_{\mathrm{x}}}}{{{\mathrm{\Phi }}_0}}} \right){\mathrm{d}}x} \right|,$$

where *L*_eff_ is the effective length of the junction along the direction of the current, accounting for the magnetic flux threading through parts of the superconducting contacts over the London penetration depths. As shown schematically in Fig. 5a, the supercurrent density has an approximately uniform distribution along the *z*-axis in Cd_3_As_2_. Thus, for a magnetic field *B*_y_ applied along *y* direction, the uniform current density results in a single-slit pattern yields the single-slit Fraunhofer pattern $\left| {{\mathrm{sin}}\left( {\frac{{\pi L_{{\mathrm{eff}}}BW}}{{{\mathrm{\Phi }}_0}}} \right)/\left( {\frac{{\pi L_{{\mathrm{eff}}}BW}}{{{\mathrm{\Phi }}_0}}} \right)} \right|$ shown in the right panel. The slight asymmetry in the Fraunhofer pattern can be due to inversion symmetry breaking in the junction which is predicted in topological semimetals^42^. In contrast, due to the surface-dominated superconductivity, the supercurrent density along the *y* direction is predominantly localized on the edges as shown in Fig. 5b. When the magnetic field is applied along *z* direction, the Fraunhofer pattern has a more sinusoidal oscillation characteristic of a SQUID pattern. Notably, the central lobe width in this case shrinks to Φ_0_ when only the top and bottom surface states dominate. This is due to the destructive interference at half flux quantum in a SQUID-like geometry.

The corresponding supercurrent distributions along the *z/y* directions (Fig. 5c, e) are obtained by transforming the single-slit pattern to the real-space current density *J*_c_(*z*) as shown, respectively, in Fig. 5d, f. The full details of the extraction procedure can be found in the Supplementary Note 13 and Supplementary Fig. 22. Clearly, for *B* applied along the *y* direction, the supercurrent spreads across the junction (Fig. 5d). On the contrary, when *B* is applied along *z* direction, the critical current envelope becomes similar to a sinusoidal oscillation in Fig. 5e. The shift towards a SQUID interference pattern corresponds to the development of sharp peaks in supercurrent density at the mesa edges. The periodicity of the critical current oscillations is contrasted to the observed Fraunhofer pattern in Supplementary Fig. 23 to prove the single period of Φ_0_ at low magnetic field. The deviation from the single slit in Fig. 5c, d may result from the fluctuations in junction length, position-dependent transparency of interface by a granularity of the Nb film and the variation of the surface carrier density. Despite the deviation, the Fraunhofer pattern is obviously different from the SQUID pattern. The first minimum of *I*_c_ at ~0.2 mT, extracted from the Fraunhofer pattern in Fig. 5c, gives the effective length of *L*_eff_ ~ 1.4 μm, which is slightly larger than the distance of two Nb electrodes. This can be explained by the triangular shape of Nb along the out-of-plane direction in the cross-section Transmission Electron Microscopy image, as shown in Supplementary Fig. 3a where the thinner Nb, close to the edge of Nb electrodes, may become non-superconducting. Besides, the effective length *L*_eff_ should also include the London penetration depth *λ*_L_ ~ 100 nm for Nb^43^ such that *L*_eff_ = *L* + 2*λ*_L_. These two reasons may contribute to the larger *L*_eff_. When the magnetic field is applied along *z*-axis, the periodicity of oscillations reaches $\frac{{{\mathrm{\Phi }}_0}}{{L_{{\mathrm{eff}}}t}}\sim 13\,{\mathrm{mT}}$ (*t* is the thickness of Cd_3_As_2_), which accords well with the SQUID pattern in Fig. 5e. We can then estimate the width of the supercurrent-carrying surface channel using a Gaussian line shape. The two surface-full regime depths *z*_s_ are extracted to be 13 and 14 nm, respectively, which is consistent with our experimental results on Andreev reflections across the Nb/Cd_3_As_2_ junctions. As control experiments, thick Cd_3_As_2_ is found to be hard to achieve Josephson effect while it performs multiple Andreev reflections (MARs) (see Supplementary Note 14 and Supplementary Figs. 24–27 for details).

## Discussion

Our experiments on the Nb/Cd_3_As_2_ and Nb/Cd_3_As_2_/Nb hybrid structures demonstrate the surface superconductivity with a large proximity gap from the parent superconductor, and a detailed supercurrent distribution is extracted from the SQI measurements. Here, we would like to discuss the physical origin of the observed surface superconductivity and its potential implications.

First, we discuss alternative explanations of the surface superconductivity, which do not require the existence of surface states, in our Cd_3_As_2_ samples. These possibilities include surface doping by charge impurities and surface band-bending effects^44,45^. While our transport and SQI measurements alone cannot directly rule out these possibilities, a rather unusual anisotropy of these effects is generally required to be compatible with the surface supercurrent distribution found in our SQI experiments. Particularly, the charge impurities or surface band-bending should occur primarily on the top and bottom surfaces, which can hardly be met by the realistic conditions of our experimental set-up. In contrast, the intrinsic physical properties of surface states (i.e. Fermi arcs), for example, their anisotropic surface distribution and thickness-independent properties, show naturally consistent characteristic with the signatures of the observed surface superconductivity (see Supplementary Note 15 and Supplementary Fig. 28 for details).

We further contrast our result with previous reports on superconductivity in surface states of a usual 3D topological insulator (TI). Topologically nontrivial surface states also exist in TI and similar BICP due to surface states has been reported in Bi_2_Se_3_/NbSe_2_ hybrid structure^46^. The contribution of surface states and bulk states in TI systems is usually separated by tuning the Fermi level within the bulk gap using electric gating or chemical doping. Generally, sample resistance is a criterion to estimate the bulk states proportion^47,48^. In Dirac semimetal Cd_3_As_2_, an effective tuning of chemical potential^32^ becomes accessible to change the transport property from bulk-dominated to surface-dominated, which is also justified by two-frequency oscillations (see Supplementary Note 9 and Supplementary Figs. 16-18 for details). Moreover, even eliminating bulk states in TI by chemical doping such as Bi_1.5_Sb_0.5_Te_1.7_Se_1.3_ system, the SQI result is different in Cd_3_As_2_ and TI because surface states cover all the surfaces of TI. Specifically, in TI, due to the uniform surface states in all surfaces, the supercurrent density is edge-dominated along all three directions which results in a mixed Fraunhofer and SQUID-like pattern rather than a pure SQUID pattern^49^. Nevertheless, the surface states are generally only on two side-surfaces along the *y*-axis of Cd_3_As_2_. This enables the observation of SQUID pattern upon magnetic fields applied along the principal *z*-axis, which is spatially resolved as edge supercurrent along the *y*-direction as shown in Fig. 5.

**Fig. 6:**
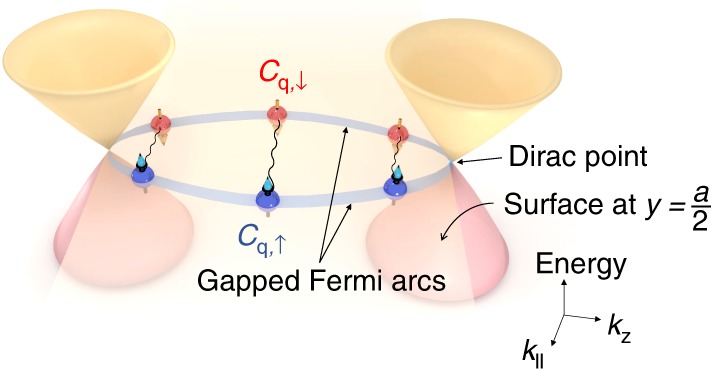
Possible superconductivity in Fermi arcs of Cd_3_As_2_. A schematic drawing of Fermi-arc superconductivity in Cd_3_As_2_. The proximity-induced *s*-wave superconductivity pairs up surface Fermi arcs with time-reversed momenta and spins. *k*_∥_ stands for (*k*_x_, *k*_y_) for the bulk and *k*_x_ for the arc, respectively

As we introduced earlier, the Fermi-arc states in Cd_3_As_2_ originate from edge states of 2D TI embedded in the Dirac semimetal. In particular, Fermi arcs labeled by momentum *k*_z_ ∈ (−*k*_0_, *k*_0_) stem from the slice of 2D TI indexed by *k*_z_. Thus, proximitizing the surface of Cd_3_As_2_ is equivalent to inducing pairing in different slices of 2D TI edges as displayed in Fig. 6. Therefore, the Cooper pairs are formed by opposite spin electrons from slices of 2D TIs with opposite *k*_z_. In other words, the surface supercurrent observed in our work can be regarded as a higher dimensional analog of edge supercurrent in 2D quantum spin Hall insulators (QSHIs). We note that with certain perturbations that break the C4-cymmetry of Cd_3_As_2_, Dirac points located along the principal axis can be gapped out. In this case, the system becomes a strong topological insulator^50^; in particular, the *k*_z_ = 0 plane is still characterized by a nontrivial Z_2_ invariant of 2D QSHIs^50^. Thus, this subtlety does not affect our conclusion that the observed surface superconductivity provides signatures of higher dimensional edge supercurrents in 2D QSHIs.

The supercurrent distribution in our surface-dominated provides the first direct evidence of surface superconductivity in topological semimetals. While the observations in the current work cannot rule out alternative physical origins other than Fermi arcs, our results call for future efforts to elucidate the possible Fermi-arc origin of the surface superconductivity. Prospectively, when a short linear Josephson junction with π phase difference is formed on the surface of Cd_3_As_2_, one pair of Majorana bound states can be created at the junction interface of each slice of 2D TI^23^. This can result in a large number of non-dispersive Majorana fermions, called Majorana flat bands, that connects the separated Dirac points located at (0, 0, ±*k*_0_)^20^. The Majorana flat bands created by superconducting Fermi arcs can be signified by a characteristic sudden jump at π phase in the Josephson current–phase relation. Similar to the single Majorana bound state at a π-junction interface on quantum spin Hall edges^23^, the Majorana flat bands on the surface of Cd_3_As_2_ are protected by time-reversal symmetry and thus remain robust against non-magnetic disorder. While evidence of single Majorana bound states and chiral Majorana edge states has been spotted in recent experiments^51,52^, experimental realization of Majorana flat bands still remains unexplored. We believe that our experimental finding of strong proximity effect on surface may establish Cd_3_As_2_ to be a new promising platform for realizing Majorana flat bands.

## Methods

### Cd_3_As_2_ nanostructure growth

The Cd_3_As_2_ nanoplates were grown using Cd_3_As_2_ powders as the precursor in a horizontal tube furnace, in which argon was a carrier gas. Before the growth, the furnace was pumped and flushed with argon several times to remove water and oxygen. The temperature was ramped to the growth temperature within 15 min, held constantly for 20 min, and then was cooled down naturally over ~2 h in a constant flow of argon before the substrates were removed at room temperature. The precursor boat was placed in the hot center of the furnace (held at 500 °C), while the smooth quartz substrates were placed in the down-stream within a very small temperature range from 200 to 350 °C. The argon flow rate is 50 SCCM (standard cubic centimeters per minute). The smooth quartz substrates then appeared shining to the naked eyes. The largest crystal plane of as-grown Cd_3_As_2_ nanoplates is [112].

### Device fabrication

The Nb/Cd_3_As_2_ hybrid structures were fabricated by electron beam lithography (EBL) technique and wet-etched by standard buffered HF solution for 5 s in the electrode regime. For Nb/Cd_3_As_2_ device, we first fabricated Cr/Au (10/150 nm) bilayers electrodes on Cd_3_As_2_ side using magneton sputtering. Then, we use standard EBL method to deposit the Nb layer. For Nb/Cd_3_As_2_/Nb Josephson junction, we use the EBL method and magnetic sputtering to deposit 140 nm-thick Nb electrodes.

### Transport measurements

Four-terminal temperature-dependent transport measurements were carried out in a Physical Property Measurement System (PPMS) system (Quantum Design) (1.9 K) and dilute refrigerator (down to 35 mK) using lock-in amplifier (SR830) and Agilent 2912. The differential conductance (d*I/*d*V*) spectra were captured by ac-modulation technique. For the PPMS measurements, lock-in amplifier provides *ac* input in series with Agilent exporting *dc* voltage. Through a large resistor (0.05–1 MΩ) the input voltage converts to a constant current. Agilent 2912 and lock-in amplifiers with a low frequency (<50 Hz) were used for d*I/*d*V* spectra measurements. The *R–T* curves were measured by d*I/*d*V* spectra while the Agilent *dc* voltage is set to zero.

### Tight-binding model of Nb/Cd_3_As_2_ transport

In the Bloch basis formed by {|*S*_1/2_, 1/2〉, |*P*_3/2_, 3/2〉, |*S*_1/2_, −1/2〉, |*P*_3/2_, −3/2〉}, the momentum-space tight-binding Hamiltonian of the Dirac semimetal Cd_3_As_2_ used in the theoretical calculations of Andreev reflections is given by^22^:

$$H_{{\mathrm{DS}}}(k) = {\it{\epsilon }}_0\left( k \right)I_{4 \times 4} + \left[ {\begin{array}{*{20}{c}} {M(k)} & {A_ + (k)} & 0 & 0 \\ {A_ - (k)} & { - M(k)} & 0 & 0 \\ 0 & 0 & {M(k)} & { - A_ - (k)} \\ 0 & 0 & { - A_ + (k)} & { - M(k)} \end{array}} \right]$$

where the matrix elements are defined as:

$$\begin{array}{l}{\it{\epsilon }}_0\left( k \right) = C_0 - E_{\mathrm{F}} + 2C_1\left[ {1 - {\mathrm{cos}}\left( {k_{\mathrm{z}}c} \right)} \right] + 2C_2\left[ {2 - {\mathrm{cos}}\left( {k_{\mathrm{x}}a} \right) - {\mathrm{cos}}(k_{\mathrm{y}}a)} \right]\\ M\left( k \right) = M_0 - 2M_1\left[ {1 - {\mathrm{cos}}\left( {k_{\mathrm{z}}c} \right)} \right] - 2M_2\left[ {2 - {\mathrm{cos}}\left( {k_{\mathrm{x}}a} \right) - {\mathrm{cos}}\left( {k_{\mathrm{y}}a} \right)} \right]\\ A_ \pm \left( k \right) = A_0\left[ {{\mathrm{sin}}\left( {k_{\mathrm{x}}a} \right) \pm i{\mathrm{sin}}(k_{\mathrm{y}}a)} \right]\end{array}$$

Here, *a* = 3 Å, *c* = 5 Å refer to the lattice constants within the *ab*-plane and along the *c-*axis, respectively. For simplicity, we neglect off-block diagonal parts which only account for higher order or bulk inversion asymmetric terms^22^. The parameters *C*_0_, *C*_1_, *C*_2_, *M*_0_, *M*_1_, *M*_2_, and *A*_0_ are set in units of eV with their values given in Supplementary Table 2 of the Supplementary Information. *E*_F_ is the Fermi level measured from the Dirac points in the Cd_3_As_2_. More details of the numerical Green’s function method for transport calculations in Fig. 4 are presented in Supplementary Note 11.

## Supplementary information

Supplementary Information

## Data Availability

The data that support the plots within this paper and other findings of this study are available from the corresponding author upon reasonable request.

## References

[1] Qi XL, Zhang SC (2011). Topological insulators and superconductors. Rev. Mod. Phys..

[2] Hasan MZ, Kane CL (2010). Colloquium: topological insulators. Rev. Mod. Phys..

[3] Bernevig BA (2015). It’s been a Weyl coming. Nat. Phys..

[4] Vafek O, Vishwanath A (2014). Dirac fermions in solids: from high-Tc cuprates and graphene to topological insulators and Weyl semimetals. Annu. Rev. Condens. Matter Phys..

[5] Armitage NP, Mele EJ, Vishwanath A (2018). Weyl and Dirac semimetals in three-dimensional solids. Rev. Mod. Phys..

[6] Wan X (2011). Topological semimetal and Fermi-arc surface states in the electronic structure of pyrochlore iridates. Phys. Rev. B.

[7] Liang T (2014). Ultrahigh mobility and giant magnetoresistance in the Dirac semimetal Cd_3_As_2_. Nat. Mater..

[8] Huang X (2015). Observation of the chiral-anomaly-induced negative magnetoresistance in 3D Weyl semimetal TaAs. Phys. Rev. X.

[9] Lv BQ (2015). Experimental discovery of Weyl semimetal TaAs. Phys. Rev. X.

[10] Xu SY (2015). Discovery of a Weyl fermion semimetal and topological Fermi arcs. Science.

[11] Xu SY (2015). Discovery of a Weyl fermion state with Fermi arcs in niobium arsenide. Nat. Phys..

[12] Xu SY (2015). Experimental discovery of a topological Weyl semimetal state in TaP. Sci. Adv..

[13] Zhang C (2016). Signatures of the Adler–Bell–Jackiw chiral anomaly in a Weyl fermion semimetal. Nat. Commun..

[14] Huang C (2018). Inducing strong superconductivity in WTe_2_ by a proximity effect. ACS Nano.

[15] Potter AC, Kimchi I, Vishwanath A (2014). Quantum oscillations from surface Fermi arcs in Weyl and Dirac semimetals. Nat. Commun..

[16] Zhang C (2017). Evolution of Weyl orbit and quantum Hall effect in Dirac semimetal Cd_3_As_2_. Nat. Commun..

[17] Wang S (2017). Quantum transport in Dirac and Weyl semimetals: a review. Adv. Phys.: X.

[18] Qi Y (2016). Superconductivity in Weyl semimetal candidate MoTe_2_. Nat. Commun..

[19] Bachmann MD (2017). Inducing superconductivity in Weyl semimetal microstructures by selective ion sputtering. Sci. Adv..

[20] Chen A, Pikulin DI, Franz M (2017). Josephson current signatures of Majorana flat bands on the surface of time-reversal-invariant Weyl and Dirac semimetals. Phys. Rev. B.

[21] Wang H (2015). Observation of superconductivity induced by a point contact on 3D Dirac semimetal Cd_3_As_2_ crystals. Nat. Mater..

[22] Wang Z (2013). Three-dimensional Dirac semimetal and quantum transport in Cd_3_As_2_. Phys. Rev. B.

[23] Fu L, Kane CL (2009). Josephson current and noise at a superconductor/quantum-spin-Hall-insulator/superconductor junction. Phys. Rev. B.

[24] He L (2016). Pressure-induced superconductivity in the three-dimensional topological Dirac semimetal Cd_3_As_2_. Npj Quantum Mater..

[25] Aggarwal L (2016). Unconventional superconductivity at mesoscopic point contacts on the 3D Dirac semimetal Cd_3_As_2_. Nat. Mater..

[26] Wang H (2016). Observation of superconductivity induced by a point contact on 3D Dirac semimetal Cd_3_As_2_ crystals. Nat. Mater..

[27] Xu Y (2014). Observation of topological surface state quantum Hall effect in an intrinsic three-dimensional topological insulator. Nat. Phys..

[28] Yilmaz T (2014). Absence of a proximity effect for a thin-films of a Bi_2_Se_3_ topological insulator grown on top of a Bi_2_Sr_2_CaCu_2_O_8_+δ cuprate superconductor. Phys. Rev. Lett..

[29] Xu SY (2014). Fermi-level electronic structure of a topological-insulator/cuprate-superconductor based heterostructure in the superconducting proximity effect regime. Phys. Rev. B.

[30] Blonder GE, Tinkham M, Klapwijk TM (1982). Transition from metallic to tunneling regimes in superconducting microconstrictions: excess current, charge imbalance, and supercurrent conversion. Phys. Rev. B.

[31] Tinkham, M. *Introduction to Superconductivity* (McGraw-Hill,1975).

[32] Moll PJW (2016). Transport evidence for Fermi-arc-mediated chirality transfer in the Dirac semimetal Cd_3_As_2_. Nature.

[33] Wilson JH (2018). Do the surface Fermi arcs in Weyl semimetals survive disorder?. Phys. Rev. B.

[34] Heslinga DR (1994). Observation of double-gap-edge Andreev reflection at Si/Nb interfaces by point-contact spectroscopy. Phys. Rev. B.

[35] Xiao X (2015). Anisotropic quantum confinement effect and electric control of surface states in Dirac semimetal nanostructures. Sci. Rep..

[36] Reyren N (2007). Superconducting interfaces between insulating oxides. Science.

[37] Saito Y (2015). Metallic ground state in an ion-gated two-dimensional superconductor. Science.

[38] Dubos P (2001). Josephson critical current in a long mesoscopic S-N-S junction. Phys. Rev. B.

[39] Hart S (2014). Induced superconductivity in the quantum spin Hall edge. Nat. Phys..

[40] Pribiag VS (2015). Edge-mode superconductivity in a two-dimensional topological insulator. Nat. Nanotechnol..

[41] Amet F (2016). Supercurrent in the quantum Hall regime. Science.

[42] Chen C (2018). Asymmetric Josephson effect in inversion symmetry breaking topological materials. Phys. Rev. B.

[43] Gubin AI (2005). Dependence of magnetic penetration depth on the thickness of superconducting Nb thin films. Phys. Rev. B.

[44] Zhang F, He Y, Chen X (2009). Guided modes in graphene waveguides. Appl. Phys. Lett..

[45] Allen MT (2015). Spatially resolved edge currents and guided-wave electronic states in graphene. Nat. Phys..

[46] Li H (2017). Origin of bias-independent conductance plateaus and zero-bias conductance peaks in Bi_2_Se_3_/NbSe_2_ hybrid structures. Phys. Rev. B.

[47] Lee S (2016). Observation of the superconducting proximity effect in the surface state of SmB_6_. Thin Films. Phys. Rev. X.

[48] Nichele F (2017). Scaling of majorana zero-bias conductance peaks. Phys. Rev. Lett..

[49] Lee JH (2014). Local and Nonlocal Fraunhofer-like pattern from an edge-stepped topological surface Josephson current distribution. Nano. Lett..

[50] Kargarian M, Randeria M, Lu Y (2016). Are the surface Fermi arcs in Dirac semimetals topologically protected?. Proc. Natl Acad. Sci. USA.

[51] Zhang H (2018). Quantized Majorana conductance. Nature.

[52] He Q (2017). Chiral Majorana fermion modes in a quantum anomalous Hall insulator–superconductor structure. Science.

