# Supplementary material for: Proximity-induced surface superconductivity in Dirac semimetal Cd3As2
Source: Nat Commun. 2019 May 17;10:2217. doi: 10.1038/s41467-019-10233-w (PMC6525265; doi:10.1038/s41467-019-10233-w)
Supplement: Supplementary file 1 — Supplementary Information [file 41467_2019_10233_MOESM1_ESM.pdf]

Supplementary Information for Proximity-induced surface superconductivity in Dirac Semimetal Cd_3As_2

Huang et al.

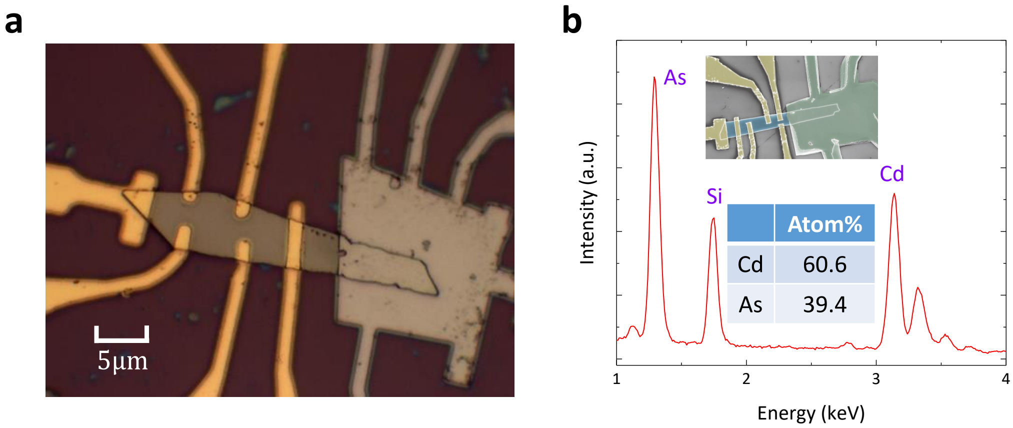

Supplementary Figure 1 | Cd_3As_2 nanoplates. (a) An optical picture of Nb/Cd_3As_2 hybrid structure (device #05). The scale bar is 5 μm. **(b)** The EDX spectrum of Cd_3As_2 nanoplate with the atomic ratio of Cd:As of 1.54: 1. The inset displays the SEM image of the device measured (device #01).

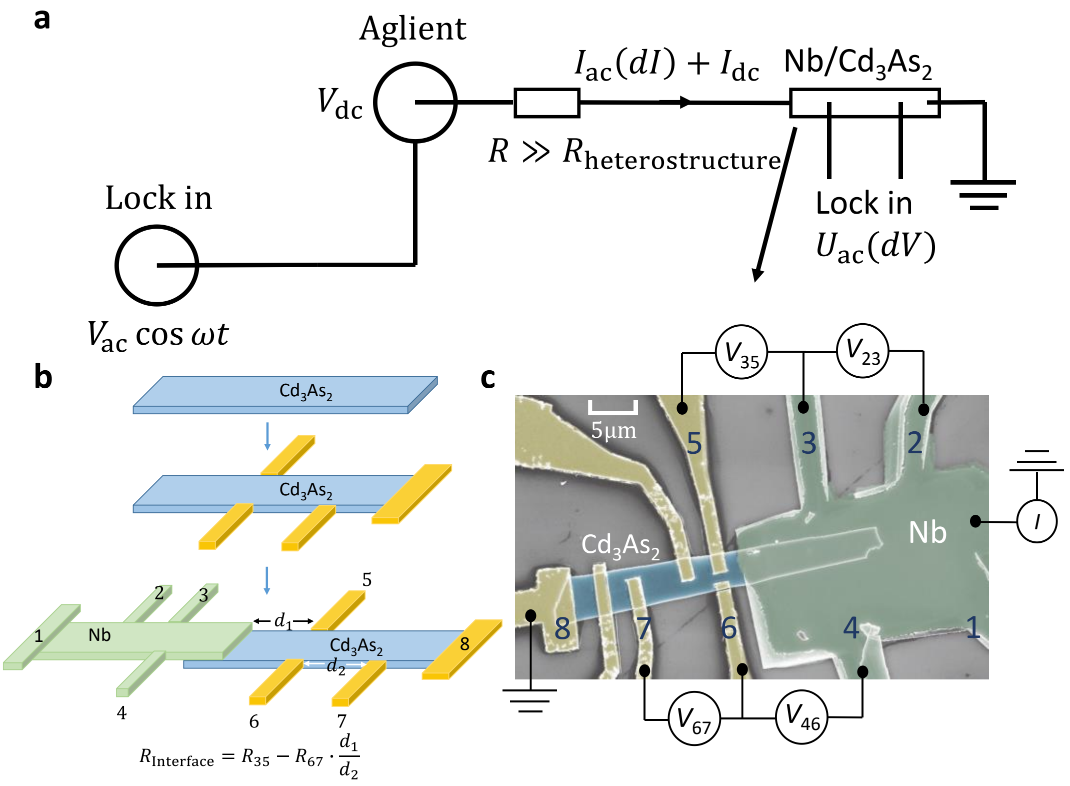

Supplementary Figure 2 | Device measurement setup. (a) A schematic diagram describing the electrical transport and the differential conductance spectroscopy measurements. **(b)** Sketch of the hybrid structure assembly process. The bottom picture shows the interface resistance measurement setup with the labeling of the electrodes in device #01. **(c)** Optical image of the hybrid structure with measurement configuration and electrode labeling. Scale bar, 5 μm.

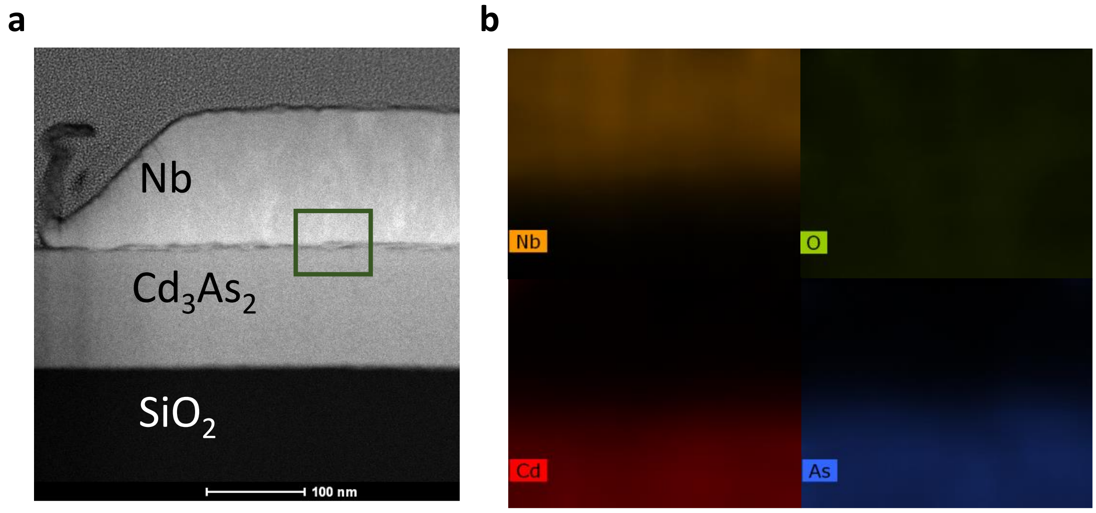

Supplementary Figure 3 | Cross-sectional TEM analysis. (a) Cross-sectional TEM image of the Nb/Cd_3As_2/Nb Josephson junction (device #06), showing 120 nm-thick Cd_3As_2 and 140 nm-thick Nb. The rectangular region is measured by EDS. Scale bar, 100 nm. **(b)** EDS mapping of Nb/ Cd_3As_2 interface. The oxygen component is ultra-low and uniform in the measured region, as an evidence of low oxidization and high interface quality.

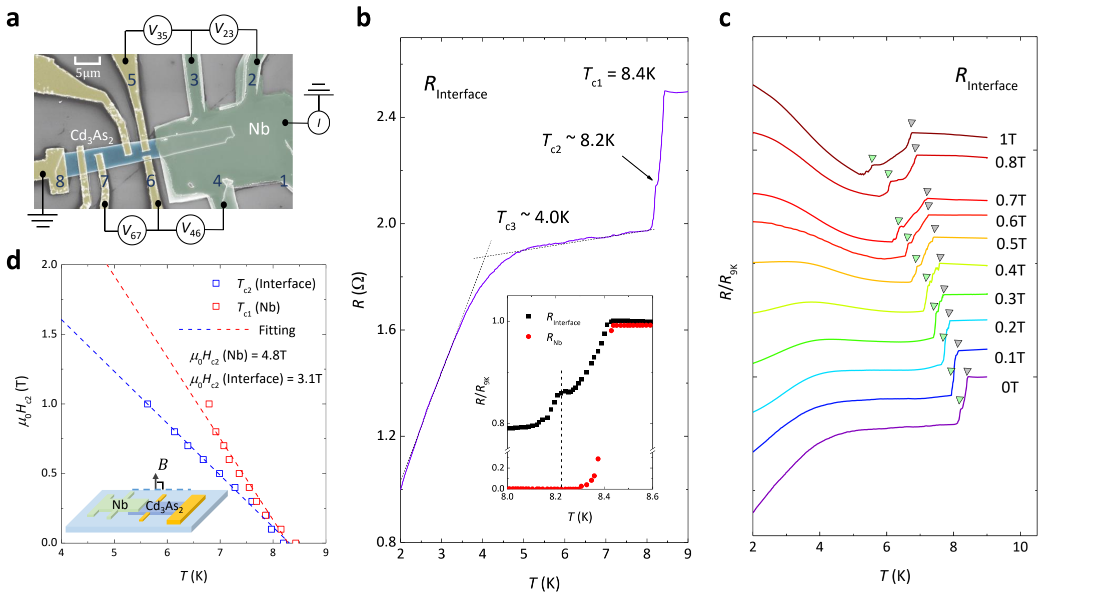

Supplementary Figure 4 | Proximity-induced superconductivity in Nb/Cd_3As_2 hybrid structure and magneto-transport characteristics. (a) False-color scanning electron microscopy image of the device with measurement configurations. Nb is deposited on top of Cd_3As_2 nanoplate. The thickness of Nb and Cd_3As_2 nanoplate is ~250 nm and ~200 nm, respectively. A four-terminal measurement across the interface was performed. A constant current (I) was applied through electrode 1 and 8 and V_{ab} is the voltage drop between electrode a and b. The voltage drop at different electrodes represents the resistance of that region. Scale bar, 5 μm. **(b)** Interface resistance-temperature curves between electrodes 3 and 5. Inset shows $R_{\mathrm{Interface}}$ and R_{Nb} at 8.0-8.6 K. The off-temperature of Nb is 8.3 K which is higher than the on-temperature of the second transition

$R_{Interface}$. **(c)** Normalized magnetic field dependent $R_{Interface}$ -*T* curves. The resistance is normalized to a normal-state value at 9 K. The curves are shifted vertically for clarity. The transition temperature is denoted by triangles. **(d)** *H*-*T* phase diagram of Nb and proximity-induced superconductivity in Cd_3As_2. To make a visual contrast, we use the on-temperature as the transition temperature. The red and blue dashed lines show the GL fitting of Nb and Cd_3As_2, respectively. Inset displays a schematic drawing with the magnetic field direction perpendicular to the Nb/Cd_3As_2 plane.

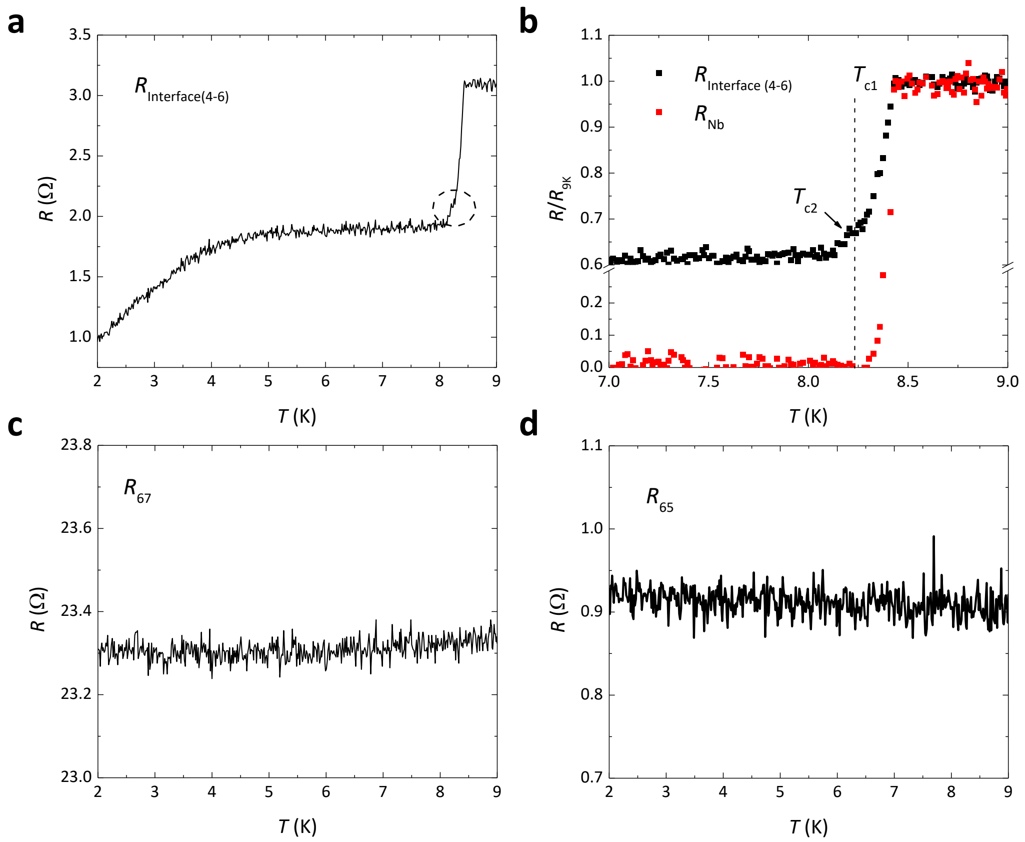

Supplementary Figure 5 | Supplementary *R*-*T* data for device #01 at 0 T. (a) Interface resistance-temperature curves measured from the other two electrodes (4-6). **(b)** The contrast of $R_{\text{Interface}}$ and R_{Nb} at 7-9 K. The off-transition temperature of Nb is 8.3 K and it is higher than the on-temperature of the second transition $R_{\text{Interface}}$. **(c)-(d)** $R_{\text{Cd}_3\text{As}_2} - T$ curves for electrodes 6-7 and 6-5, respectively.

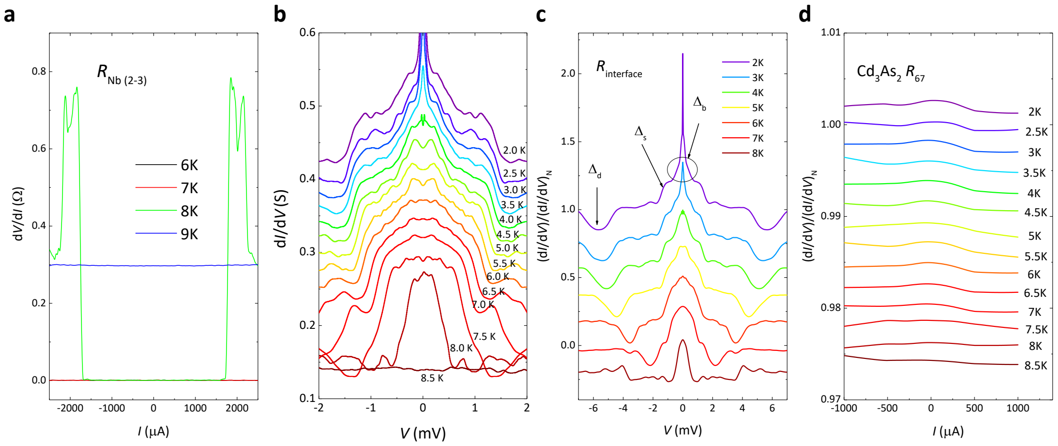

Supplementary Figure 6 | Supplementary differential conductance data for device #01. (a) Differential resistance of R_{23} at 6-9 K. **(b)** Temperature dependent d*I*/d*V* of interface conductance. (**c**) Zero-field temperature-dependent d*I*/d*V* of $R_{Interface}$, normalized by the normal-state conductance at 9 K. The curves are vertically shifted for clarity. Four obvious features are observed as the above-gap dip (Δ_d), bias-independent conductance plateau (BICP) (Δ_s), broad peak (Δ_b) from proximity-induced superconductivity in Cd_3As_2 bulk states and zero-bias conductance peak (ZBCP). **(d)** Temperature-dependent normalized differential conductance spectra of Cd_3As_2.

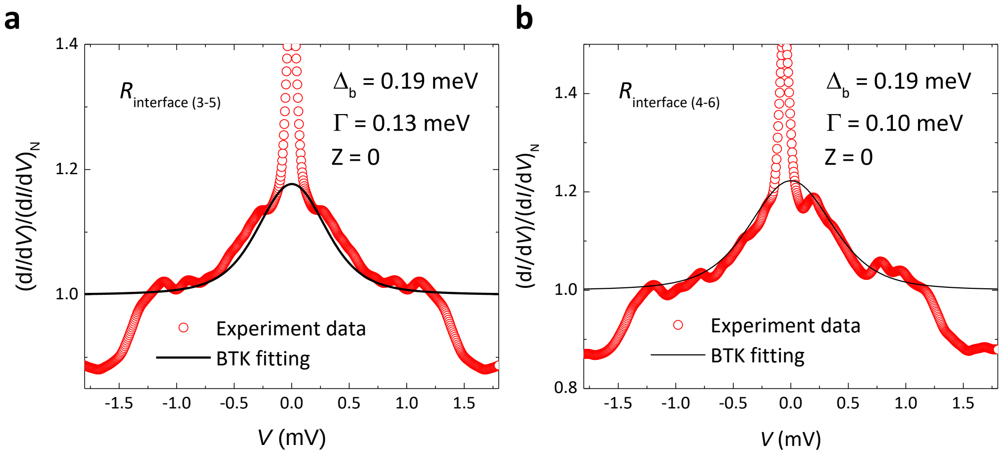

Supplementary Figure 7 | BTK fits of differential conductance spectra in Nb/Cd_3As_2 device #01. (a)-(b) Normalized d*I*/d*V*-*V* characteristics from experimental data (red hollow circles) and fitting results (black solid line) at 2 K and 0 T for $R_{Interface}$ from different electrodes.

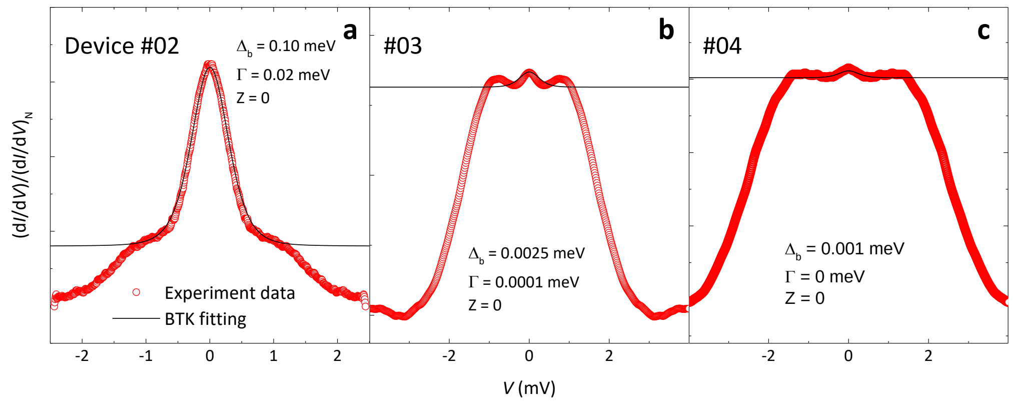

Supplementary Figure 8 | BTK fitting of d*I*/d*V* in device #02, #03 and #04 at 2K.

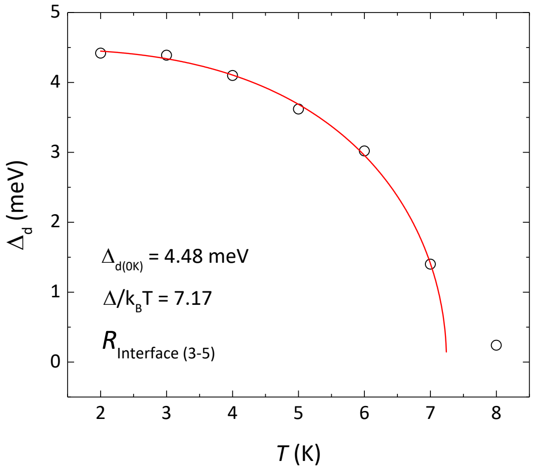

Supplementary Figure 9 | BCS Fits of the superconducting gap $\Delta_d - T$ relation in Nb/Cd_3As_2 device #01.

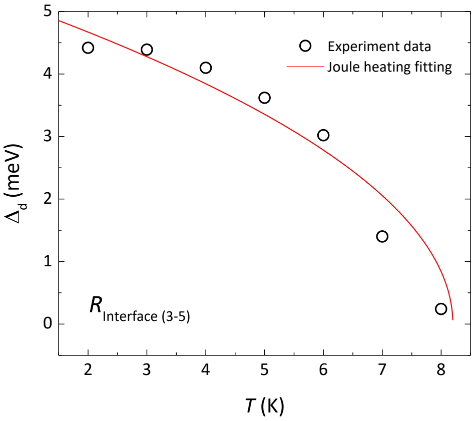

Supplementary Figure 10 | Joule heating fit of $\Delta_d \sim \sqrt{T_c - T}$ relation in Nb/Cd_3As_2 device #01. Black hollow circle dots are experiment data and red solid line is the fitting curve.

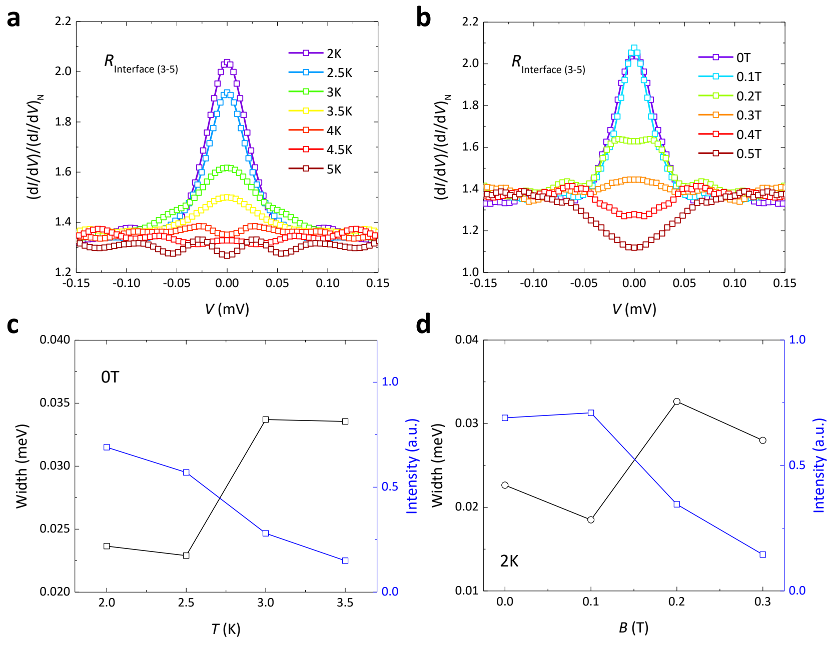

Supplementary Figure 11 | ZBCP in Nb/Cd_3As_2 device #01. (a) Differential conductance spectra at temperatures from 2 to 5 K under zero magnetic field. **(b)** Differential conductance spectra at different magnetic fields and 2 K. **(c)** The ZBCP peak width (black open squares) and intensity (blue open squares) as a function of temperatures at zero magnetic field. Solid lines are guides to the eyes. The width is determined by the standard full width at half maximum (FWHM). **(d)** The peak width (black open squares) and intensity (blue open squares) as a function of magnetic field at 2 K. Solid lines are guides to the eyes.

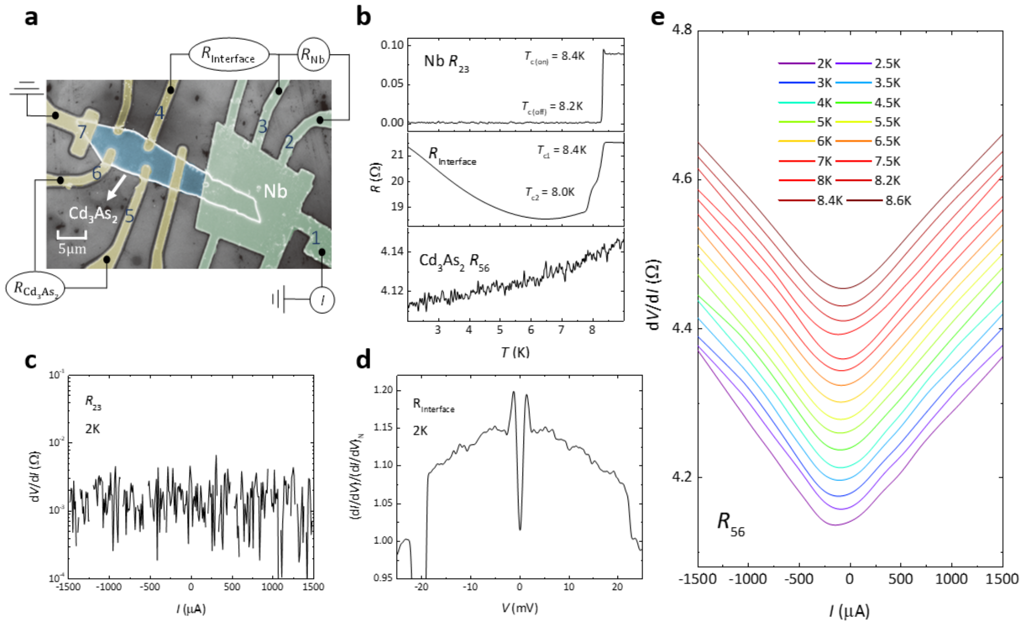

Supplementary Figure 12 | Transport properties in device #05. (a) False-color SEM image of the device with measurement configurations. Scale bar, 5 μm. **(b)** *R*-*T* curves in Nb, interface, and Cd_3As_2. **(c)** Differential resistance of R_{23} at 2 K. **(d)** Normalized differential conductance curve of $R_{\text{Interface}}$ at 2 K. **(e)** Temperature-dependent differential resistance spectra of R_{56}.

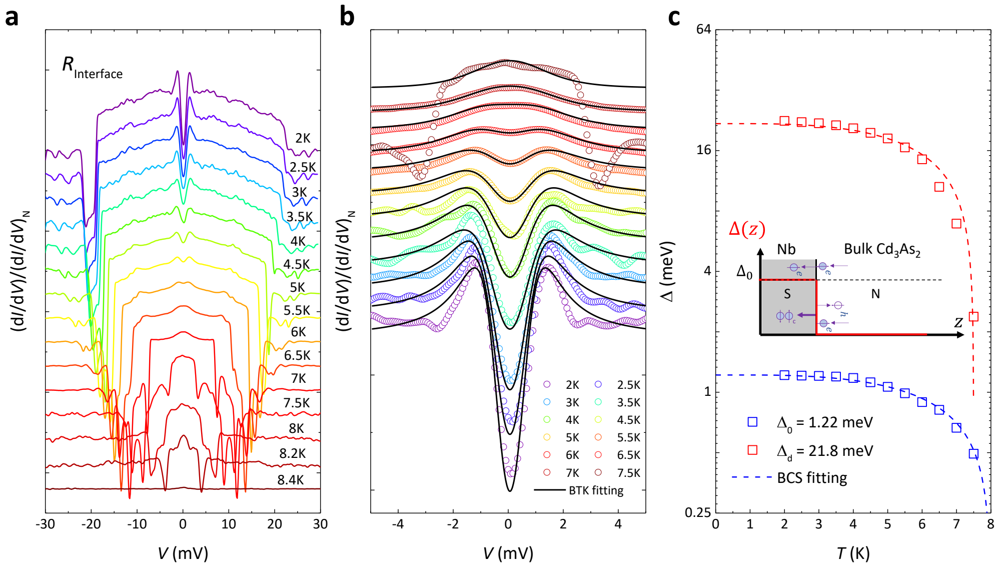

Supplementary Figure 13 | Temperature-dependent d*I*/d*V* spectra of $R_{\mathrm{Interface}}$. (a) Full-range d*I*/d*V* spectra at 2-8.4 K. **(b)** BTK fits of the conductance dip with two peaks region. **(c)** BCS fit of the temperature-dependent gap. Dashed lines are the fitting curves. Inset shows a schematic drawing of the hybrid structure in bulk-dominated Cd_3As_2 with no proximity-induced superconductivity-quasiparticle scattering occurring at the interface.

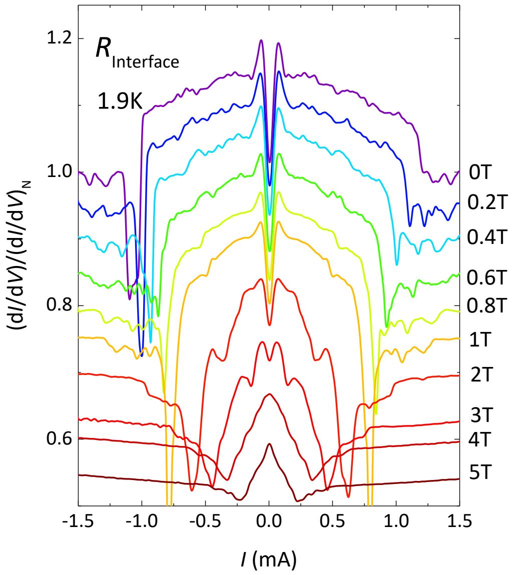

Supplementary Figure 14 | Magnetic field dependent d*I*/d*V* spectra of $R_{\mathrm{Interface}}$ at 1.9 K.

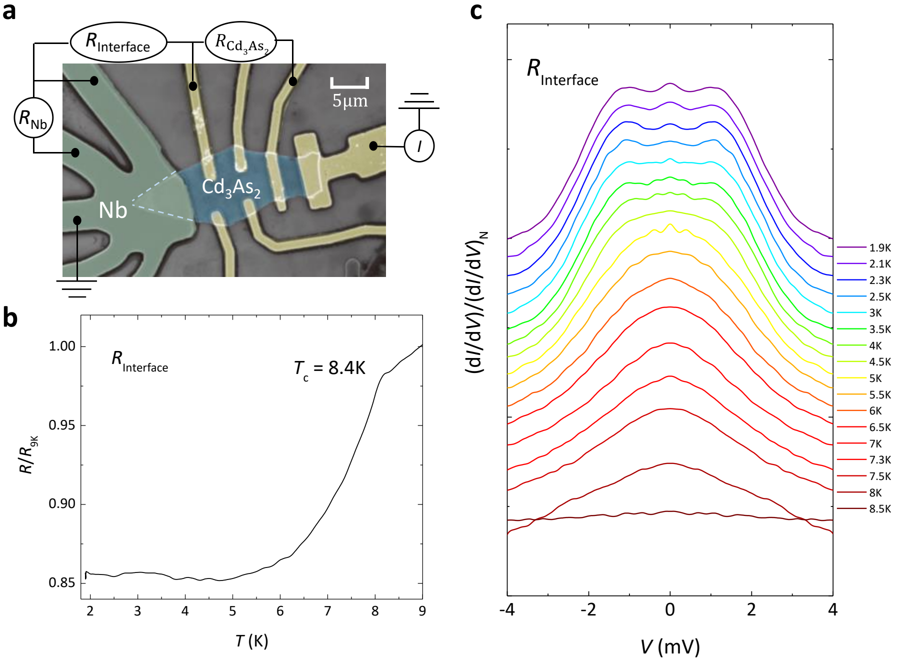

Supplementary Figure 15 | Supplementary data for Nb/Cd_3As_2 device #03. (a) False-color SEM image of the device with the measurement configuration. Scale bar, 5 μm. **(b)** Normalized *R*-*T* curve of $R_{\text{Interface}}$ at zero magnetic field. **(c)** Temperature-dependent d*I*/d*V* spectra.

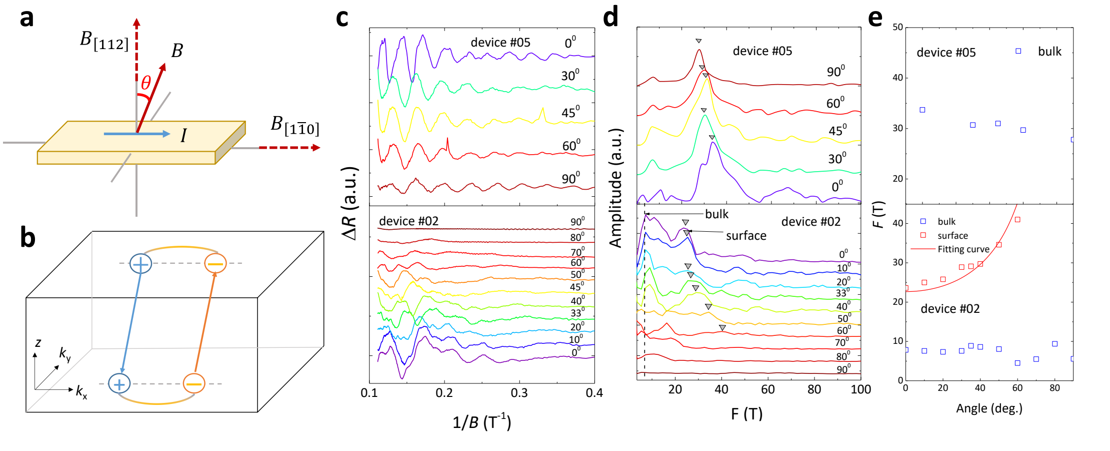

Supplementary Figure 16 | Formation of Weyl orbits and 2D Fermi surface in Cd_3As_2 nanoplates (device #05 and #02). (a) Illustration of the field direction with respect to the device geometry. **(b)** Sketch of Weyl orbits in Cd_3As_2 in a thin slab. The orbit involves both the Fermi-arc surface states connecting the Weyl nodes of opposite chirality, and the bulk states of fixed chirality. **(c)** Angle-dependent MR in Cd_3As_2 nanoplates (device #05 and #02). **(d)** Comparisons of the angle-dependent FFT spectra of quantum oscillations in device #05 and #02, respectively. **(e)** Angle dependence of oscillation frequency in device #05 and 02. The red curve is a fitting curve for 2D Fermi surface.

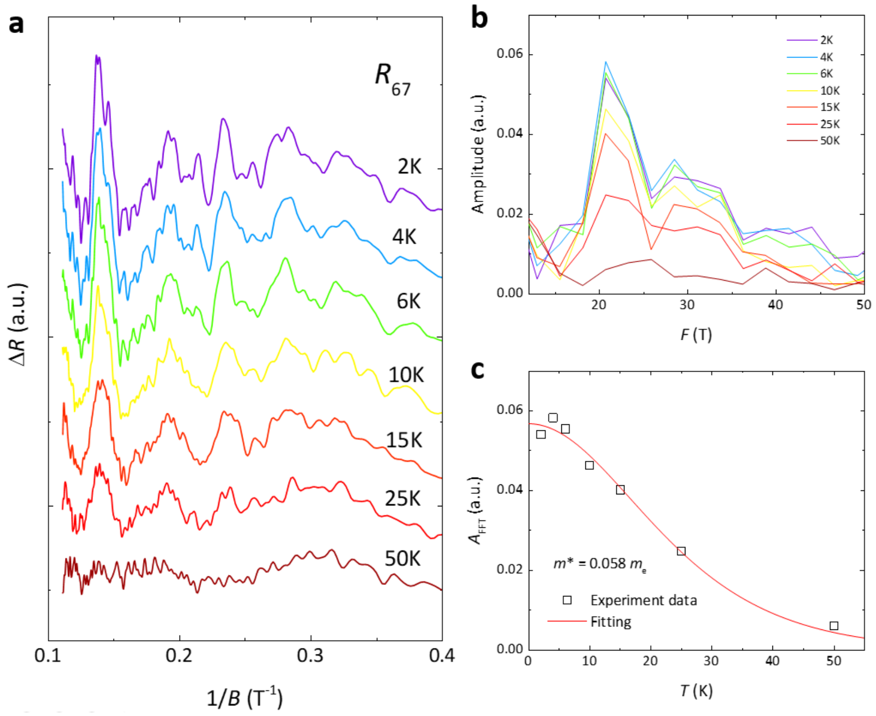

Supplementary Figure 17 | SdH oscillations in Cd_3As_2 with both surface and bulk states in device #01. (a) Temperature dependence of extracted SdH oscillations. **(b)** The FFT spectra at various temperatures. The temperature dependence of FFT amplitude and fitting of effective mass **(c)** Temperature dependence of the quantum oscillation amplitude for $F_B = 20.5$ T.

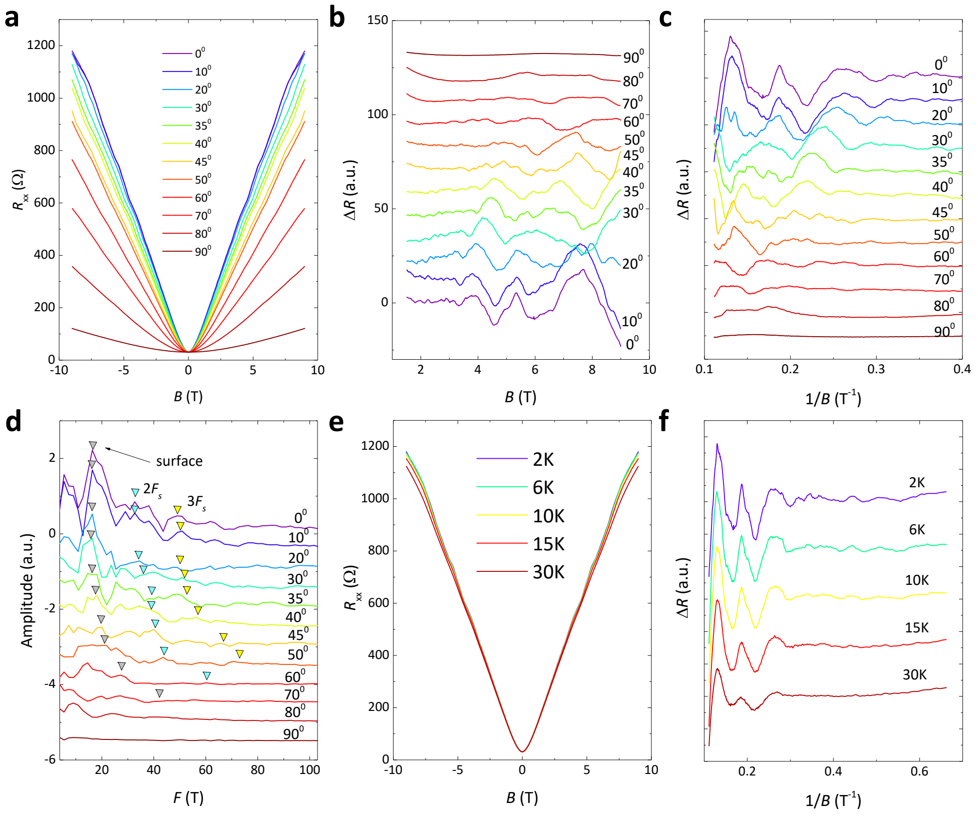

Supplementary Figure 18 | Surface-dominated SdH oscillations in Cd_3As_2 nanoplate in device #03. (a) The field dependence of magnetoresistance R_{xx} at different angles at 2 K. **(b)** The angle dependence of extracted SdH oscillations (ΔR) in device #03 **(c)** The angle dependence of ΔR versus $1/B$. **(d)** Angle dependence of FFT spectra with bulk and surface frequency indicated by the triangles. **(e)** Temperature dependence of R_{xx} under perpendicular magnetic fields. **(f)** Temperature dependence of extracted SdH oscillations.

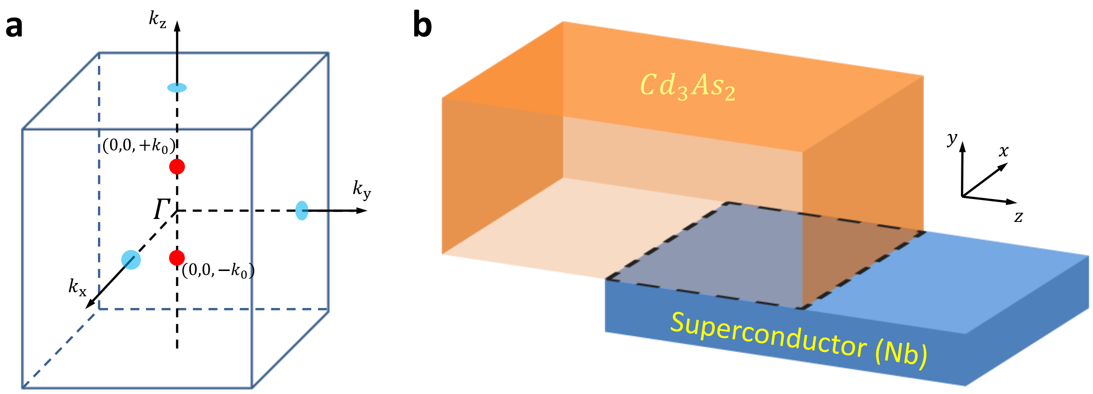

Supplementary Figure 19 | Schematics for Cd_3As_2/superconductor junction. (a) Schematic figure of the 3D Brillouin zone of Cd_3As_2. Dirac points located at $(0,0,\pm k_0)$ along the [001]-axis are indicated by the red dots. **(b)** Schematic of the junction formed by Cd_3As_2 and Nb. The real-space coordinates are defined in the same way as the k-space coordinates in (a). The interfacial area is enclosed by the black dashed-line.

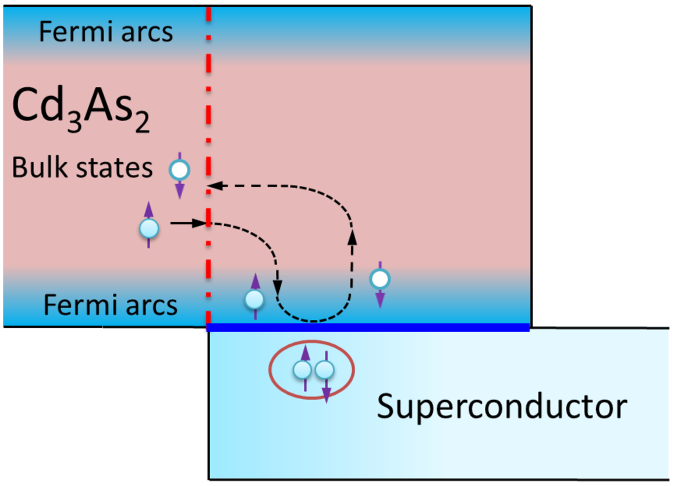

Supplementary Figure 20 | A schematic drawing of the Nb/Cd_3As_2 hybrid structure. Blue solid line indicates the physical interface between Dirac semimetal and the superconductor. Red dashed line indicates the interface chosen to calculate the differential conductance. By current conservation law, all possible Andreev reflections at the NS interface (blue line) are captured by the scattering matrix defined at the artificial interface (red dashed line).

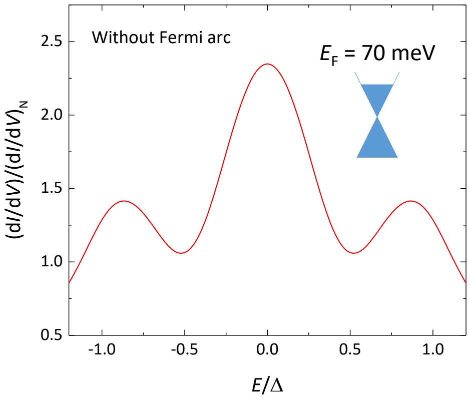

Supplementary Figure 21 | Numerical calculations of the normalized Andreev reflection amplitude in Cd_3As_2/Nb junction. The chemical potential lying above the Dirac points ($E_F =$ 70 meV) in the absence of Fermi-arc states.

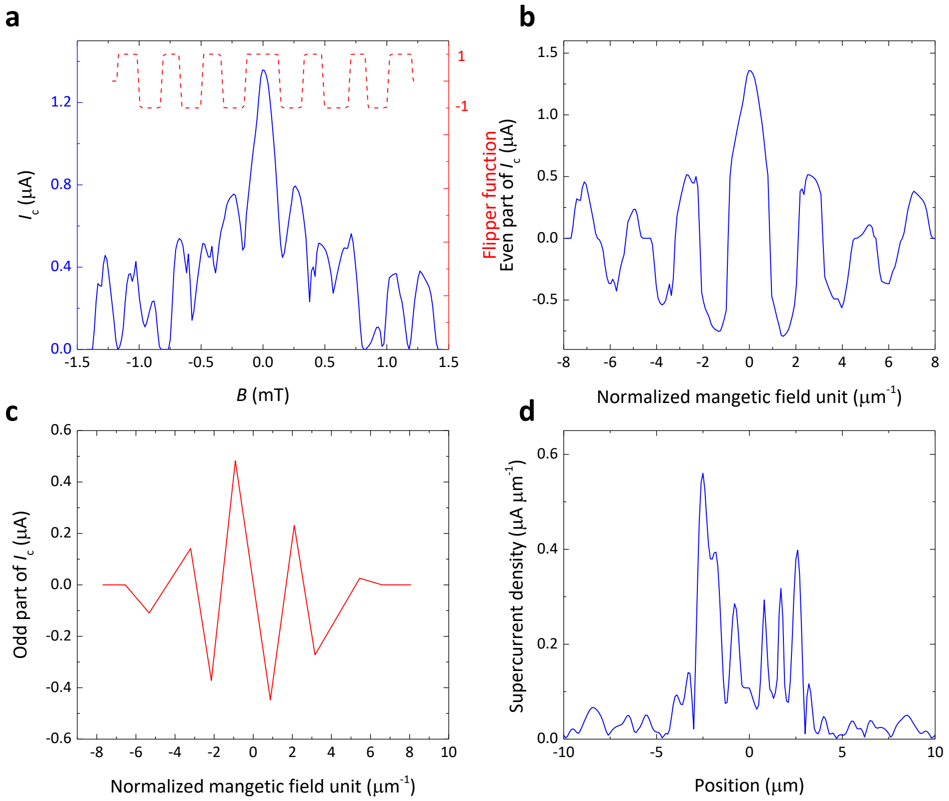

Supplementary Figure 22 | Analysis of current density profile in device #06. (a) Recovering the critical current phase. When the current distribution is mostly symmetric, the experimentally observed critical current envelope (blue line) approaches zero between peaks. In such cases, a flipping function (red dashed line) that changes sign at each node of the envelope enables the recovery of $J_C(B)$ from $I_c^{max}(B)$. **(b)** The recovered critical current $I_E(\beta)$ that corresponds to the even part of the current density profile $J_E(z)$. **(c)** The recovered critical current $I_O(\beta)$ that corresponds to the odd part of the current density profile $J_O(z)$. **(d)** The current density profile $J_S(z)$.

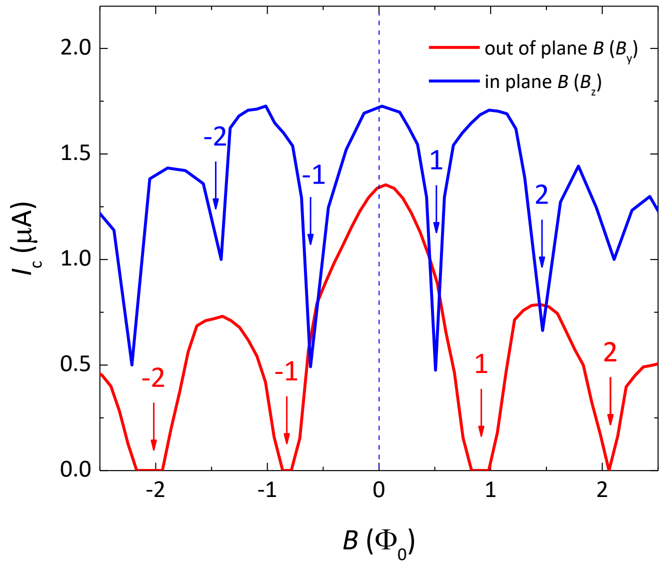

Supplementary Figure 23 | Critical current modulation by magnetic fields. In order to compare the dependencies (*e.g.*, the positions of the minima), the magnetic field scale has been normalized to Φ_0 to make it easy to contrast the width of B in the first order and other orders.

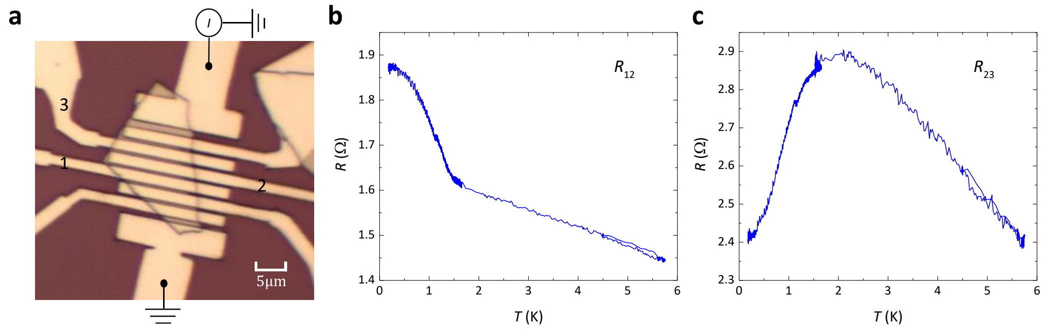

Supplementary Figure 24 | *R*-*T* properties of Josephson junction device #07. (a) Optical microscopy image of the device with $12\ \mu m$ width and $400\ nm$ length channel. Scale bar, $5\ \mu m$. **(b)-(c)** The *R*-*T* curve for R_{12} and R_{23}.

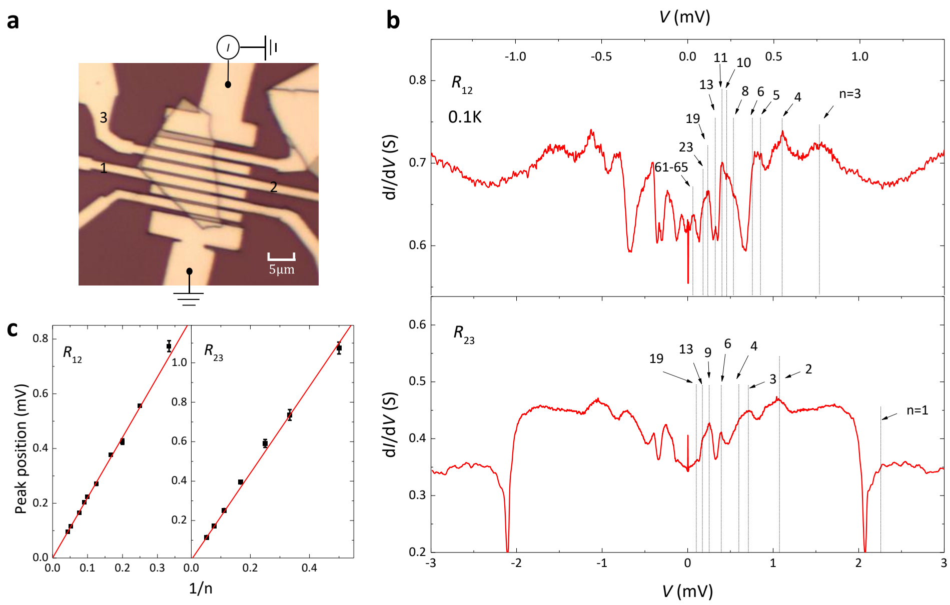

Supplementary Figure 25 | Multiple Andreev reflections in thick Cd_3As_2 Josephson junctions. **(a)** The optical image of the device #07. Scale bar, 5 μm. **(b)** The d*I*/d*V* versus *V* data for R_{12} and R_{23} taken in a four-probe method. Arrows and dashed lines mark the theoretical MARs peak positions at $V_n = \frac{2\Delta}{ne}$ with values of n indicated. **(c)** Plot of the MARs peak position versus the inverse index $1/n$. The red line is a linear fit to all the data points, and the slope is proportional to the energy of the gap. Error bars of the black dots are added based on both experimental resolution.

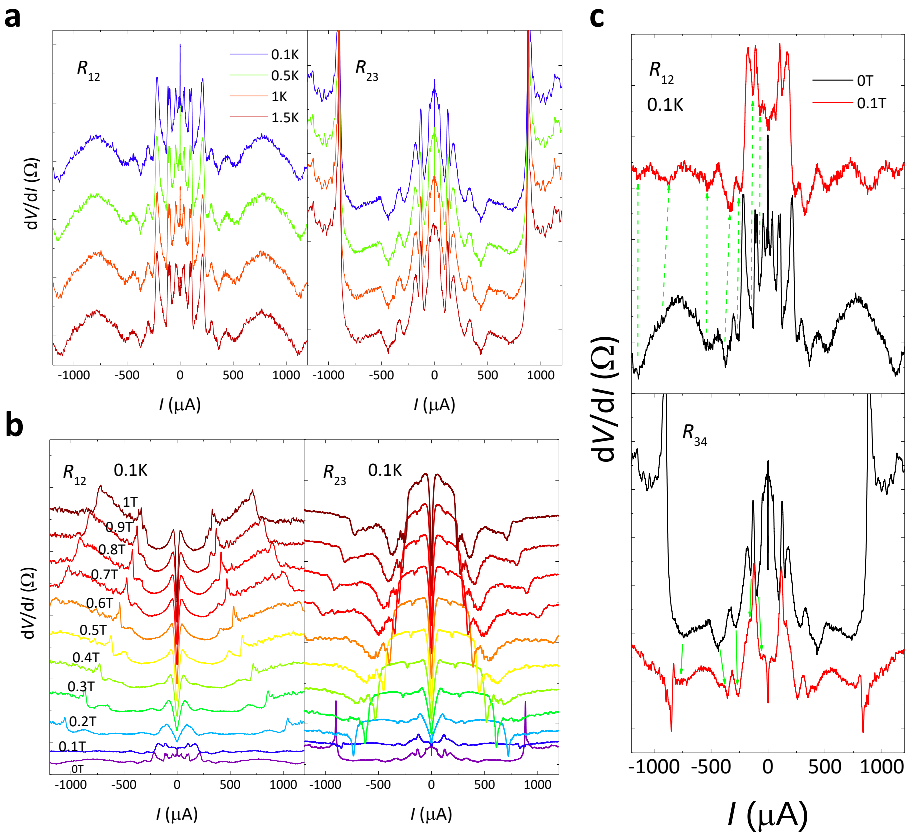

Supplementary Figure 26 | Temperature and magnetic field dependent dV/dI. (a) Temperature-dependent dV/dI under zero magnetic field. **(b)** Magnetic field dependent dV/dI properties at 0.1 K. **(c)** The reappearance of first and second MARs signal under 0.1 T magnetic field of R_{12}.

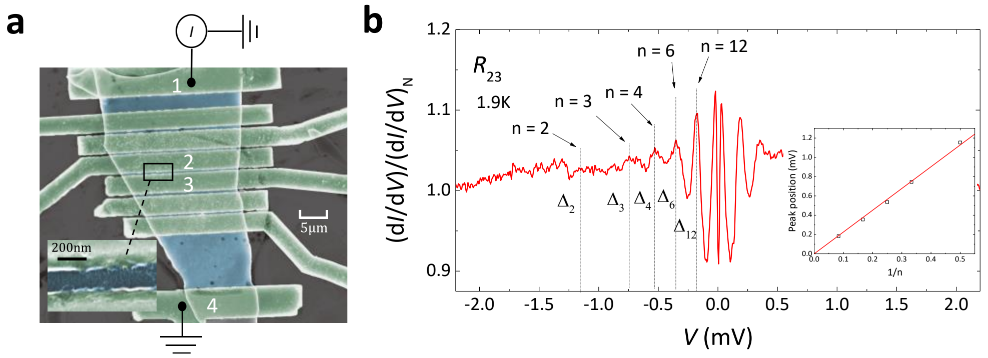

Supplementary Figure 27 | MARs in another thick Cd_3As_2 Josephson junction (device #08). (a) Device structure and SEM image with 200 nm length junction. **(b)** Normalized dI/dV spectra at 1.9 K of R_{23} indicating MARs with several peaks. Inset shows a plot of the MARs peak position versus the inverse index $1/n$. The red line is a linear fit to all the data points, and the slope is proportional to the energy of the gap.

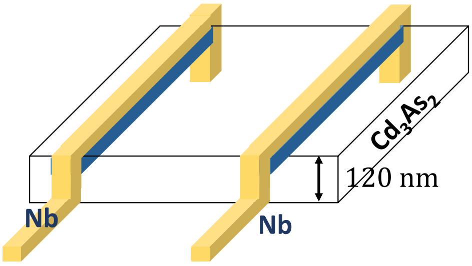

Supplementary Figure 28 | Possible impurity induced region. The two Nb electrodes are deposited as displayed in the yellow region. The area shown in blue which is close to the contact interface should have the highest possibility with induced impurities.

Supplementary Table 1 | Estimated band parameters in Cd_3As_2 at 2K

Device	Thickness (nm)	F_B (T)	F_S(T)	A_S/A_B	v_F (10^5m/s)	$m^*(m_e)$
#01	~ 200	20.5	30.8	0.57	~4.97	0.058
#02	~ 200	~7.9	23.6	0.76	~2.75	0.063
#03	< 150	~4.7	15.8	1.83	~2.76	0.050
#04	< 150	~3.0	12.3	2.2	~2.01	0.055
#05	> 300	33.0	/	0	~4.89	0.075
Moll *et al.*[32]	/	36.5	61.5	/	/	

The effective mass m^* and Fermi velocity v_F can be extracted from the SdH oscillations.

Supplementary Table 2 | Parameters for the Hamiltonian H_{DS}. All parameters set in units of *eV*.

C_0	C_1	C_2	M_0	M_1	M_2	A_0
−0.001	0.0011	0.0665	−0.2706	−0.3194	−0.8239	1.4855

Supplementary Note 1. The growth of Cd_3As_2 nanostructures

Supplementary Fig. 1a is an optic image of Nb/Cd_3As_2 hybrid structure after transferring onto SiO_2/Si substrate (device #05 for thick Cd_3As_2). Nanoplates with a typical thickness around 100-250 nm were chosen to study the proximity-induced superconductivity in Fermi arcs while the samples with a thickness exceeding 300 nm are for bulk-dominated study. A thickness less than 150 nm can enhance the phase coherence of Fermi arcs and surface contribution while avoiding the gap opening due to the quantum confinement effect. To identify the composition of the sample, energy dispersive X-ray spectroscopy (EDX) has been carried out as shown in Supplementary Fig. 1b and the result is reproducible. The atomic ratio of Cd and As is $1.54:1$. The slight composition deviation might be due to arsenic vacancies[1].

Supplementary Note 2. Device fabrication and measurement setup

The Nb/Cd_3As_2 hybrid structures were fabricated as shown in Supplementary Fig. 2b. We first transfer the as-grown Cd_3As_2 nanoplates onto SiO_2/Si substrate. We used a standard electron beam lithography (EBL) technique to deposit Cr/Au (10/150 nm) bilayer electrodes on Cd_3As_2 using magneton sputtering. Then, another EBL process is performed to fabricate the Nb film on top of Cd_3As_2.

The differential conductance spectra were captured by an *ac*-modulation technique using a lock-in amplifier (SR830) and Agilent 2912 as shown in Supplementary Fig. 2a. A small *ac* voltage coupled with a *dc* input was fed through a large standard resistor (0.05-1.0 MΩ) in series to deliver a constant *dc* current, which was superimposed by a small *ac* current. This current is passed through the device. The lock-in amplifier working at 17 Hz measures the *ac* voltage as the differential voltage $\mathrm{d}V$; the $\mathrm{d}I$ is estimated to be a constant since the change of device resistance is four orders of magnitude smaller than the resistor. By scanning the *dc* current I, we can acquire $\frac{\mathrm{d}V}{\mathrm{d}I}-I$ curves. Then *ac* voltage is integrated by $V=\int\frac{\mathrm{d}V}{\mathrm{d}I}\mathrm{d}I$, and $\frac{\mathrm{d}I}{\mathrm{dV}}$ is plotted against V to produce the differential conductance spectrum.

All the measurements are deployed by the four-terminal method to prevent contact resistance. For example, the measurement setup for device #01 is presented in Supplementary Fig. 2b and c. We apply a constant current from electrode 1 to 8, and meanwhile, measure the voltage difference between electrode 2 and 3 as the Nb resistance (by dividing the constant current). Such a four-terminal method can exclude the effect of current redistribution in the 2-terminal method. To make the measurement more accurate, all the resistance data (R_{23}, R_{35}, R_{46}, R_{56} and R_{67}) are measured at the same time.

We can estimate the interface resistance $R_{\mathrm{Interface}}$ as shown at the bottom of Supplementary Fig. 2b. The measured R_{35} contains contributions from Nb, interface and Cd_3As_2. The values of the individual contribution cannot be precisely estimated. But we can reasonably calculate the interface resistance by $R_{\mathrm{Interface}}=R_{35}-R_{67}\cdot\frac{d_1}{d_2}$, where we subtract the contribution of the Cd_3As_2 resistance from the interface to electrode 5. The Nb resistance is small enough. Thus, the obtained junction resistance

is a valid estimation.

Then, we performed the cross-section TEM experiments of Nb/Cd_3As_2 interface as shown in Supplementary Fig. 3a, the thickness of each layer can be estimated to be 140 nm and 120 nm for Nb and Cd_3As_2, respectively. Moreover, the EDS mapping result displayed in Supplementary Fig. 3b indicates low oxidization and high interface quality.

Supplementary Note 3. *R*-*T* measurement for device #01

Temperature-dependent resistance (*R*-*T*) curves at $T \geq 2.0$ K across the junction are shown in Supplementary Fig. 4b. The resistance drop region can be broadened as the applied magnetic field increases (Supplementary Fig. 4c) which makes it easier to distinguish the two transitions. When the temperature is lower than 4 K (T_{c3}), the resistance drops again and a small magnetic field of 0.5 T can destroy the transition. The upper critical fields H_{c2}(Nb) and H_{c2}(Interface), as a function of temperature, are plotted in Supplementary Fig. 4d. The coherence length of Nb is extracted to be $\xi_{GL} = 10$ nm by Ginzburg-Landau (GL) model[2] $\mu_0 H_{c2}^{\perp} = \frac{\Phi_0}{2\pi\xi_{GL}(0)^2}(1 - \frac{T}{T_c})$, as shown by red dashed line, where Φ_0 represents the flux quantum. The coherence length is much smaller than the thickness of the Nb film, which excludes the reduced superconducting order parameter by the normal-state Cd_3As_2. Linear relation *H*-*T* curve indicates a critical field of 3.1 T.

From Supplementary Fig. 2b, we can simultaneously measure $R_{\text{Interface}}$ of electrodes 3-5 and electrodes 4-6. Similarly, three transitions are observed in Supplementary Fig. 5a-b. The difference of the drop for the first transition is due to the different Nb resistance between $R_{\text{Interface}\,(4-6)}$ and $R_{\text{Interface}\,(3-5)}$. Besides, from the resistance measurements of two sides Cd_3As_2 as shown in Supplementary Fig. 5c-d, we can exclude the possibility of Cd_3As_2 intrinsic effect to the three transitions[3].

Differential conductance curves were acquired as shown in Supplementary Fig. 6. The critical current of Nb is $I_c \sim 1700$ μA at 8 K in Supplementary Fig. 6a which is larger than our measurement limit at $T \leq 7$ K, indicating that Nb is always in the superconducting state in our d*I*/d*V* measurement. Supplementary Fig. 6b shows the original data of interface differential conductance. Since the normal state conductance does not change with bias at 8.5 K, we use the conductance (~0.4 S) at 8.5 K to normalize the d*I*/d*V*. Supplementary Fig. 6c shows the d*I*/d*V*-*I* curve of $R_{\text{Interface}(3-5)}$. Supplementary Fig. 6d demonstrates the d*I*/d*V* curves of Cd_3As_2 without AR-related signals. Thus, we can conclude that the behavior including ZBCP, BICP, ZBBP and above-gap dip in $R_{\text{Interface}}$ is due to the interface superconductivity.

Now, we need to rule out other possibilities of the ZBBP. Our measurements show a proximity-induced gap appearing immediately below T_c with 0.14 times the normal conductance, different from previous reports on phase conjugation[4]. The device with high interface barrier Z shows the lack of the proximity effect and only the superconducting gap of Nb is observed, thus dismissing other possible processes occurring in Nb alone (Supplementary Fig. 12).

Supplementary Note 4. BTK fits of Nb/Cd_3As_2 differential conductance spectra

This section mainly focuses on the theoretical calculations to understand the density of states (DOS) features at the Nb/Cd_3As_2 interface with the superconducting proximity effect at an energy scale within the intrinsic superconducting gap. We used BTK theory to simulate the differential conductance at finite temperature with respect to the bias voltage V, that is given by[5]

$$\frac{\mathrm{d}I_{\mathrm{NS}}}{\mathrm{d}V}(V) = I_0 \frac{\mathrm{d}}{\mathrm{d}V} \times \int_{-\infty}^{+\infty} [f(E-eV,T) - f(E,T)]\sigma(E)\mathrm{d}E, \quad (1)$$

where $f(E,T)$ is the Fermi distribution function. $\sigma(E)$ demonstrates the BTK conductance at $T = 0$ as follows,

$$\sigma(E) = \tau_{\mathrm{N}} \frac{1+\tau_{\mathrm{N}}|\gamma(E)|^2 + (\tau_{\mathrm{N}}-1)\left|\gamma(E)^2\right|^2}{|1+(\tau_{\mathrm{N}}-1)\gamma(E)^2|^2}, \quad (2)$$

where τ_{N} is transparency of the barrier in the BTK approximation of current injection totally perpendicular to the Nb/Cd_3As_2 interface:

$$\tau_{\mathrm{N}} = \frac{1}{1+Z^2}, \quad (3)$$

$Z = \frac{H}{\hbar v_{\mathrm{F}}}$ is a dimensionless parameter modeled with a δ-function barrier $V = H\delta(x)$. $\gamma(E)$ is a complex function,

$$\gamma(E) = \frac{N_{\mathrm{q}}(E)-1}{N_{\mathrm{p}}(E)}, \quad (4)$$

where $N_{\mathrm{q}}(E) = \frac{E}{\sqrt{E^2-\Delta^2}}$ and $N_{\mathrm{p}}(E) = \frac{\Delta}{\sqrt{E^2-\Delta^2}}$, whose real parts are the BCS quasiparticle and pair density of states, respectively.

In addition, we consider the quasiparticle lifetime. The AR structures in the experimental spectra are not only depressed in amplitude but also spread in energy which is attributed to the reduction of the quasiparticle lifetime, resulting from the imaginary part of the quasiparticle self-energy, as discussed in the tunneling regime by Dynes *et al* [6], and inelastic quasiparticle scattering processes[7]. It is possible to globally take these effects into account by including in the BTK model a single broadening parameter Γ in the form of an imaginary part of the energy $E \rightarrow E + \mathrm{i}\Gamma$ which is called modified BTK model.

We summarize the BTK fits in the broad peak region in Supplementary Fig. 7. Interface differential conductance spectra measured by two different electrodes, $R_{\mathrm{Interface\,(3-5)}}$ and $R_{\mathrm{Interface\,(4-6)}}$, are shown in Supplementary Fig. 7a and b with fitting parameters $\Delta = 0.19$ mV, $\mathrm{Z} = 0$ and $\Gamma = 0.13$ mV and 0.10 mV, respectively. The data is fitted well and zero Z value indicates a high interface quality. Corroborated with our theoretical simulations in Supplementary section 7, we attribute it to the proximity effect on the bulk Cd_3As_2 states.

The BTK fits of dI/dV in three devices (#02, #03, #04) are plotted in Supplementary Fig. 8. We use the broad peak region to prove that the behavior is attributed to the proximity-induced superconductivity.

Supplementary Note 5. BCS fits of the superconducting gap

Here, we use an approximate equation of BCS theory to fit the data[8, 9]

$$\Delta(T) = \Delta_0 \tanh(1.74\sqrt{\frac{T_c}{T} - 1}), \tag{5}$$

with $\Delta_0 = 1.76 k_B T_c$ as the temperature dependence of the pair potentials. Under magnetic field, $T_c(B) = T_c(0\ \mathrm{T})\sqrt{1 - \frac{H}{H_{c2}}}$, then the magnetic field dependent superconducting gap can be approximately expressed by[2, 8]

$$\Delta \propto \sqrt{1 - \frac{H}{H_{c2}}} \tanh(1.74\sqrt{\frac{T_c}{T} \cdot \sqrt{1 - \frac{H}{H_{c2}}} - 1}), \tag{6}$$

The temperature-dependent above-gap dip is shown in Supplementary Fig. 9.

Supplementary Note 6. Above-gap dip analysis

The above-gap dip shows a good BCS relation (Supplementary Fig. 9) with temperature and it disappears above T_c, indicating its physical origin from the superconductivity of Nb. The above-gap dip has been observed in many systems[10, 11, 12, 13] and still under debate. Applying a high bias during the differential conductance measurement may produce Joule heating which causes a break-down of superconductivity in Nb and drives the resistance to the normal state superimposed by irregular structure[11]. The above-gap dips at Δ_d are caused by a breakdown of a small excess current observed in the superconducting state. Δ_d has a temperature dependence given by $\sqrt{T_c - T}$ corresponding to an energy balance condition, where the dissipated power

$$P = \frac{\Delta_d^2}{R_{\mathrm{Interface}}} \propto \sqrt{T_c - T}, \tag{7}$$

is proportional to the temperature shift[10].

We plot the $\Delta_d \sim \sqrt{T_c - T}$ relation in Supplementary Fig. 10 and use the Joule heating model to fit. The fitting is not good, where Δ_d has saturated around 4 K in experiments rather than increased with a large value in the fitting curve. Such a saturation behavior is more suitable for the local destruction of superconductivity. We note that the above-gap dip occurs at a current of ~ 2 mA, corresponding to a current density of $j \sim 5 \times 10^3\ \mathrm{A/cm^2}$. Such a large j could destroy superconducting pairing near the Nb/Cd_3As_2 interface and affect the local contact resistance[14] near the interface[12]. Indeed, device #03 (no proximity effect) also exhibits an above-gap dip at large bias ~ 20 meV, corresponding to a large current density of $j \sim 4 \times 10^3\ \mathrm{A/cm^2}$, similar to device #01. Thus, we attribute Δ_d to depairing near the Nb/Cd_3As_2 interface which affects the local contact resistance[14] or the local destruction of superconductivity of the Nb near the interface[12].

Supplementary Note 7. ZBCP analysis

Figure S11a shows the measured differential conductance spectra at different temperatures within a smaller bias range of ± 0.15 mV. The ZBCP is pronounced at 3.5 K and becomes stronger and sharper as temperature decreases. The emergence of the ZBCP coincides with the third resistance drop observed in *R*-*T* curves as explained in the main text. This means that the ZBCP is associated with the proximity-induced superconductivity or AR in Cd_3As_2 nanoplate. The temperature dependence of the ZBCP intensity and width is plotted in Supplementary Fig. 11c. The ZBCP intensity is increased from 3.5 to 2 K. More interestingly, the ZBCP width is almost constant below 2.5 K, even decreases a little at low temperatures. With increasing the magnetic field, the width of the ZBCP is a bit broadened and the intensity is reduced. When the magnetic field is above 0.3 T, the ZBCP cannot be observed.

The ZBCP has been reported in various superconductor-normal metal hybrid structures[15, 16, 17, 18, 19], but its physical origin is still under debate. The ZBCP can be induced by the proximity-induced pair current across Schottky barrier at superconductor-semiconductor interfaces[4]. But the ZBCP width is expected to increase with decreasing temperatures, which is contrary to the behavior from our experiments and no barrier is observed in Nb/Cd_3As_2.

The second possible mechanism is related to incoherent accumulation of AR, which happens when there is a large probability of backscattering due to the involvement of the other surface of the normal-metal thin film[13, 16]. ZBCP of this kind usually increases immediately below T_c which contradicts to our results.

The third possibility is related to coherent scattering of carriers near the interface due to a phase conjugating between the electron’s and the hole’s trajectories, leading to an enhanced AR probability[15]. A ZBCP caused by this mechanism is sensitive to both temperature and magnetic field, since it involves a coherent loop. However, it should begin to appear in differential conductance spectra right below T_c while in our devices the ZBCP can only be observed at 3.5 K. Besides, these theories do not take into account the strong spin-orbit coupling (SOC) and its resulted Berry phase[12]. In the presence of strong SOC, the phase accumulated by the incident electron along its path cannot be canceled by the retro-reflected hole. Furthermore, the theory[20] suggests that this kind of ZBCP often appears in junctions with a relatively strong scattering rate, and that the value of the conductance peak will not exceed the conductance of the normal state, whereas in our experiments the ZBCP can be 1.5 times larger than the normal state conductance. Therefore, we believe that the ZBCP is not caused by the aforementioned constructive interference.

The fourth scenario is to attribute ZBCP to a pair current flowing between the superconducting Nb and the proximity-induced superconducting Cd_3As_2 phase. Its behavior will resemble the critical supercurrent of a Josephson junction. As the temperature decreases, the critical current of a Josephson junction will first increase and then gets saturated. For a ZBCP of this type, therefore, its peak width is expected to increase with decreasing *T*. However, The ZBCP width does not change much in Supplementary Fig. 9c and the pair current picture seems inapplicable to our results.

The fifth probability is Andreev bound state of an anisotropy superconductor (*s*-

wave or *d*-wave)[21, 22, 23] or the Majorana zero mode in the core of the vortices of topological superconductor[19]. ZBCP of these kinds usually shows a weak dependence on the magnetic field. However, this is apparently not our case. The ZBCP is quenched quickly by 0.4 T magnetic field in Supplementary Fig. 11b.

Some situations like Kondo correlations and weak anti-localization are also not suitable to explain our results because the ZBCP we observed is related to superconductivity.

The sixth case is the proximity-induced superconductivity in another Cd_3As_2 band. Note that the projection onto [112] direction can make the Fermi surface anisotropic. Another Cd_3As_2 band projected onto [112] direction may account for the observed ZBCP with a lower transition temperature (T_{c3}) and gap (Δ_b').

The seventh mechanism of ZBCP involves unconventional superconductivity with an asymmetric orbital order parameter[24, 25]. This mechanism has been proved in *p*-wave superconductor Sr_2RuO_4[24], superconductor/topological insulator[12] and proximity-induced *p*-wave superconductivity in graphene[26]. Since Cd_3As_2 has topological non-trivial order in bulk states, by taking into account the unconventional phase-diagram in Fig. 1d, a possible theoretical mechanism has been reported in Cd_3As_2 that quasi-2D helical *p*-wave superconducting states exist in the bulk[27] with specific k_z, which is explainable for the appearance of the ZBCP in this experiment.

Supplementary Note 8. Supplementary data for Nb/Cd_3As_2 device #03 and #05

Here we discuss the transport properties in bulk-dominated Cd_3As_2 device #05. The SEM image in Supplementary Fig. 12a illustrates clear white color Cd_3As_2 boundary, indicating its large thickness. Figure S12b shows *R*-*T* curves of Nb, interface, and Cd_3As_2, respectively[3]. Superconducting transition begins at 8.4 K and finishes at 8.2 K similar to device #01. Two transitions occur at $T_{c1} = 8.4$ K and $T_{c2} = 8.0$ K. The first corresponds to Nb superconducting. The second is unclear at this moment, because the resistance increases at low temperature, indicating superconducting tunneling behavior. As a contrast, $R_{Cd_3As_2}$ does not show any transition. Figure S12c-d display d*I*/d*V* spectra of Nb, interface and Cd_3As_2, respectively. Nb is in the superconducting state with the applied current and Cd_3As_2 shows no particular behavior. Two signatures can be easily observed in $R_{\mathrm{Interface}}$ which are above-gap dips at $\Delta_d \sim \pm 20$ mV and a conductance dip around zero bias with two pronounced conductance peaks at $\Delta_d \sim \pm 1.3$ mV, consistent with the superconducting gap of Nb. Since the AR can enhance the conductance below the superconducting gap and the tunneling effect happens in a non-transparent interface, the conductance dip with two peaks is attributed to AR and superconducting tunneling. Unlike normal metals in which various bands cross the Fermi level, only two bands[28, 29] take part in transport in Cd_3As_2 and such a low density of electrons cannot sustain a BICP.

Then, we discuss the temperature and magnetic field dependence of d*I*/d*V* spectra. Both two features are temperature dependent (Supplementary Fig. 13a) and can be well fitted by BCS theory (Supplementary Fig. 13c). Moreover, the conductance dip with two peaks region can be well fitted by BTK model in Supplementary Fig. 13, showing the evidence of AR with a parameter $Z = 1.25$. Furthermore, the magnetic field

dependent dI/dV spectra in Supplementary Fig. 14 confirm that both two features are attributed to superconductivity-related effect. As discussed in Supplementary section 4.3, we think that the above-gap dip is due to the local destruction of superconductivity of Nb near the interface because it does not change with the different bulk or surface contribution in Cd_3As_2.

The supplementary data for surface dominated Cd_3As_2 device #03 is displayed in Supplementary Fig. 15. Suplementary Fig. 15b shows R-T curves of $R_{\mathrm{Cd_3As_2}}$. Only one transition at $T_{\mathrm{c1}} = 8.4$ K is observed. Contrasting with dI/dV spectra in Supplementary Fig. 15c, the BICP corresponds to the transition in R-T curve. Besides, a small ZBCP emerges at $T_{\mathrm{c1}} = 3.0$ K, due to the proximity-induced superconductivity in bulk states. The ZBCP is too small to be detected in the R-T curve and a small resistance drop at ~3 K is on the same order as the background noise. The resistance saturates at low temperature and the Andreev reflection amplitude from bulk states is much weaker than surface channels. The gap from BTK fits at 2K in Supplementary Fig. 8b-c is also quite small. Even we estimate the proximity-induced bulk gap at zero temperature by BCS theory that $\Delta(2\ \mathrm{K}) = \Delta_0 \tanh(1.74\sqrt{\frac{3}{2} - 1}) = 0.85\Delta_0$, Δ_0 does not change much. Therefore, we can conclude the weak proximity effect in bulk states.

Supplementary Note 9. Formation of Weyl orbits and 2D Fermi surface in Cd_3As_2 nanoplates

The simple bulk band structure and the controllable thickness growth of nanoplates with different Fermi levels make Cd_3As_2 a good candidate for studying the Fermi-arc states through SdH oscillations[1, 30, 31, 32]. As shown in Supplementary Fig. 16a, we apply a constant current in Cd_3As_2 nanoplates along $[1\bar{1}0]$ direction. Perpendicular and parallel magnetic fields correspond to $[112]$ and $[1\bar{1}0]$ direction, respectively. Figure S16b illustrates the formation of Weyl orbits and Fermi arcs in thin Cd_3As_2. Figure S16c shows a typical angle dependence of MR curves in bulk-dominated and bulk-surface-mixed Cd_3As_2. Clear beating patterns are observed in the SdH oscillations in device #02, indicating possible multiple cyclotron orbits.

The behavior is in stark contrast with the single-frequency SdH oscillations observed in bulk-states dominated device #05. The angle-dependence of FFT spectra is shown in Supplementary Fig. 16d. Two peaks are found in device #02 while only one distinct peak exists in device #05. Both bulk peaks do not change much at different angles. However, the other peak position increases with a larger angle. The angle dependence of the surface frequency can be well fitted by $1/\cos\theta$ function in Supplementary Fig. 16e, corresponding to the 2D Fermi surface. Nevertheless, the second frequency has also been suggested due to the Fermi surface nesting[33] or band curvature near the Lifshitz transition[34] in bulk states. Notably, the bulk states in both samples show a similar oscillation frequency, indicating very close Fermi levels in these devices, which excludes the influence of band structure difference at different Fermi levels[1].

To estimate the superconducting coherence length in device #01, we need to

calculate the diffusion coefficient D, which constitutes two parameters k_{F}, v_{F} that can be extracted from the SdH oscillations. We show the SdH oscillations in device #01 in Supplementary Fig. 17. Following the Lifshitz-Kosevich formula[35], the oscillation component ΔR_{xx} can be described by

$$\Delta R_{\mathrm{xx}} \propto R_{\mathrm{T}} R_{\mathrm{D}} R_{\mathrm{S}} \cos 2\pi(\frac{F}{B}+\gamma), \tag{8}$$

where R_{T}, R_{D} and R_{S} are three reduction factors accounting for the phase smearing effect of temperature, scattering and spin splitting, respectively. Figure S17a demonstrates the temperature-dependent SdH oscillations extracted from MR. To fundamentally understand the Fermi level, we extract the bulk oscillation frequency $F_{\mathrm{b}} = 20.5$ T. We can obtain the cross-section area of the Fermi surface A_{F} from the relation $F = \left(\frac{\phi_0}{2\pi^2}\right) A_{\mathrm{F}}$, where $\phi_0 = \frac{h}{2e}$. As the Fermi surface is isotropic[36], the Fermi vector of k_{F} can be extracted from $k_{\mathrm{F}} = \sqrt{A_{\mathrm{F}}/\pi} = 0.0249\text{Å}^{-1}$.

Temperature-dependent oscillation ΔR_{xx} can be captured by the temperature smearing factor $R_{\mathrm{T}} \propto \frac{2\pi^2 k_{\mathrm{B}} T m^*/\hbar e B}{\sinh(2\pi^2 k_{\mathrm{B}} T m^*/\hbar e B)}$, where k_{B} is the Boltzmann's constant, $\hbar$ is the reduced Plank's constant and m^* is the average cyclotron effective mass. Temperature dependent FFT spectra is displayed in Supplemetnary Fig. 17b. By performing the best fit of the thermal damping oscillation to the equation by FFT amplitude in Supplementary Fig. 17c, the effective mass is extracted to be $m^* = 0.058\, m_{\mathrm{e}}$, where m_e is electron mass. The Fermi velocity v_{F} can be extracted by $v_{\mathrm{F}} = \frac{\hbar k_{\mathrm{F}}}{m^*} = 4.97 \times 10^5 \mathrm{m/s}$. The mean free path of Cd_3As_2 bulk could be estimated by $l_{\mathrm{MFP}} = \frac{\sigma h}{2e^2 k_{\mathrm{F}}} \sim 190$ nm.

Since the thickness of Cd_3As_2 is large enough to be treated as a 3D material, the electron diffusion coefficient D is given by $D = \frac{v_{\mathrm{F}} l_{\mathrm{MFP}}}{3}$, where v_{F} is Fermi velocity. In device #01, $D = 3.15 \times 10^{-2} \mathrm{m^2 s^{-1}}$ and the coherence length in the clean limit ($\xi_{\mathrm{N}} \ll l_{\mathrm{MFP}}$)

$$\xi_{\mathrm{N}} = \frac{\hbar v_{\mathrm{F}}}{k_{\mathrm{B}} T}, \tag{9}$$

is 452 nm at 8.4 K, the superconducting transition temperature of Nb. The obtained ξ_{N} is large than l_{MFP} in all low temperature regime ($T < 8.4$ K), thus we use the dirty limit expression

$$\xi_{\mathrm{N}} = \sqrt{\frac{\hbar D}{k_{\mathrm{B}} T}} = \sqrt{\frac{\hbar v_{\mathrm{F}} l_{\mathrm{MFP}}}{2 k_{\mathrm{B}} T}}, \tag{10}$$

ξ_{N} is 207 nm at 8.4 K, seemingly not to accord with the dirty limit. However, when the temperature is lower, the ξ_{N} can be larger than l_{MFP} (at 2 K, $\xi_{\mathrm{N}} = 424$ nm). We can then use this value as a good estimation.

The SdH oscillations in device #03 are illustrated in Supplementary Fig. 18. The analysis is similar, and the parameters are summarized in Supplementary Table 1.

Supplementary Note 10. Bulk and surface contribution to Andreev reflection

We note that the 1.14 times conductance enhancement in Fig. 1b cannot be used to judge the interfacial Z for surface channels. This is because the normal-state conductance ($T > T_c$) of our hybrid junction always has contributions from the bulk states, not just from the surface channels alone.

Suppose the conductance from the bulk states and surface states are G_{B} and G_{S} at normal states, respectively. The conductance at non-superconducting regime can be expressed as $G_{\mathrm{N}} = G_{\mathrm{B}} + G_{\mathrm{S}}$. At 8.2 K where only the surface states participate in AR, the whole conductance becomes,

$$G_{8.2\ \mathrm{K}} = 2G_{\mathrm{S}} + G_{\mathrm{B}} = 1.15G_{\mathrm{N}}, \tag{11}$$

Then, we get the bulk and surface contribution $G_{\mathrm{B}} = 0.85G_{\mathrm{N}}$ and $G_{\mathrm{S}} = 0.15G_{\mathrm{N}}$. The surface depth proportion is $\frac{z_{\mathrm{S}}}{d}\sim 0.1-0.2$ which indicates that the contribution is reasonable. At 2 K, the proximity effect results in a d_{S}-thick superconducting layer in Cd_3As_2 and the bulk resistance should be $\frac{1}{1.26}\left(1-\frac{d_{\mathrm{S}}}{d}\right)\frac{1}{G_{\mathrm{B}}}$ as we consider the AR enhancement. The conductance at 2 K can be expressed as

$$G_{2\ \mathrm{K}} = 2G_{\mathrm{S}} + 1.26\frac{d}{d-d_{\mathrm{S}}}G_{\mathrm{B}} = 2.22G_{\mathrm{N}}, \tag{12}$$

where $d\sim 1.5\ \mu\mathrm{m}$ is the distance between the electrode and the interface. $d_{\mathrm{S}}\sim 0.5\ \mu\mathrm{m}$ is solved from $G_{2\ \mathrm{K}}$. Such a superconducting layer is comparable to the coherence length ξ_{N} which also verifies our semi-quantification model. Therefore, the large conductance enhancement at low temperatures is due to the enhancement of proximity-induced superconducting layer.

Supplementary Note 11. Theoretical simulations

In this section, we present the detailed tight-binding Hamiltonian used in our theoretical calculations. The total Hamiltonian for the Nb/Cd_3As_2 junction can be written as:

$$H_{\mathrm{tot}} = H_{\mathrm{DS}} + H_{\mathrm{SC}} + H_{\mathrm{c}}, \tag{13}$$

where $H_{\mathrm{DS}}/H_{\mathrm{SC}}$ refers to the Hamiltonians for the Dirac semimetal Cd_3As_2, and the superconductor (Nb) respectively. H_{c} is the coupling Hamiltonian at the Dirac semimetal/superconductor interface.

The tight-binding Hamiltonian H_{DS} for Cd_3As_2 is defined in the Method section of the main text. The model parameters are listed in Supplementary Table 2.

The superconducting Nb is modeled by an *s*-wave superconductor with square lattice geometry. In the Nambu basis $\Psi_{\boldsymbol{k}} = \left(\psi_{\mathbf{k},\uparrow}, \psi_{\mathbf{k},\downarrow}, \psi^{\dagger}_{-\mathbf{k},\uparrow}, \psi^{\dagger}_{-\mathbf{k},\downarrow}\right)^{T}$, the bulk Hamiltonian for the superconductor is given by:

$$\mathcal{H}_{\mathrm{SC}} = \sum_{k} \Psi^{\dagger}_{\mathrm{k}} H_{\mathrm{SC}}(\mathbf{k}) \Psi_{\mathrm{k}}, \tag{14}$$

$$H_{\mathrm{SC}}(\mathbf{k}) = \left[2t\left(\cos(k_{\mathrm{x}}a) + \cos\left(k_{\mathrm{y}}a\right) + \cos(k_{\mathrm{z}}a)\right) - \mu_{\mathrm{S}}\right]\sigma_0\tau_{\mathrm{z}} + \Delta\sigma_{\mathrm{y}}\tau_{\mathrm{y}}, \tag{15}$$

Here, t refers to the hopping amplitude, μ_s is the chemical potential in the superconductor, and Δ is the mean-field *s*-wave pairing strength. In Fig.3, we set $t = -0.8$ eV, $\mu_{\mathrm{s}} \approx -3t$, $\Delta = 5$ meV.

To construct a numerically solvable model for the junction formed by Cd_3As_2 and Nb, in our simulations we consider the junction geometry shown schematically in Supplementary Fig. 19, which is physically equivalent to the junction geometry defined

in the main text. For simplicity, we choose the [010] surface of Cd_3As_2 to interface with the top surface of the superconductor. Since the Dirac points are located at $(0,0,\pm k_0)$ along the [001]-axis in the Brillouin zone of Cd_3As_2, with this choice there exists Fermi arc states on the [010] surface which can couple directly to the superconductor. In addition, the periodic boundary condition is assumed along the *x*-direction, and momentum k_{x} can serve as a good quantum number.

With the junction geometry defined in Supplementary Fig. 19b, the coupling Hamiltonian H_c at the junction interface can be written as:

$$H_{\mathrm{c}}(k_{\mathrm{x}}) = \sum_{\langle \mathbf{R},\mathbf{R}'\rangle, s\sigma} t_{\mathrm{c}}\psi^{\dagger}_{\mathbf{R}',\sigma}(k_{\mathrm{x}})c_{\mathbf{R},s\sigma}(k_{\mathrm{x}}) + h.c.$$

$$= \sum_{m\in\mathrm{Interface}}\sum_{\mathrm{s},\sigma} t_{\mathrm{c}}\psi^{\dagger}_{(\mathrm{m},\mathrm{N}_{\mathrm{z}}'),\sigma}(k_{\mathrm{x}})c_{(\mathrm{m},1),\mathrm{s}\sigma}(k_{\mathrm{x}}) + h.c, \qquad (16)$$

Here, $c_{\mathbf{R},\mathrm{s}\sigma}(k_{\mathrm{x}})$ annihilates an electron at site **R** in Cd_3As_2 with orbital $s(=\mathrm{S},\mathrm{P})$, spin $\sigma(=\uparrow,\downarrow)$ and momentum k_{x}. In Fig.4, we set the coupling strength to be $t_{\mathrm{c}} \cong t$.

To calculate the differential conductance $G_{\mathrm{c}} = \mathrm{d}I/\mathrm{d}V$ in the transport measurements, we consider a semi-infinite Cd_3As_2 with a section of its bottom layer attached to the top layer of the superconductor as shown schematically in Supplementary Fig. 20.

We note that for voltage bias $V < \Delta/\mathrm{e}$, the only scattering process contributing to current flow is the Andreev reflection, in which an incoming electron is converted into a reflected hole. While this process occurs at the physical interface (blue solid line in Supplementary Fig. 20) formed by Cd_3As_2 and Nb, current conservation guarantees that the current flow driven by Andreev reflections can be obtained from scattering matrices defined at any interface in the Cd_3As_2. To calculate the conductance in a convenient way, we choose the interface indicated by the red dashed-line in Supplementary Fig. 20 in our numerical calculations. The $\mathrm{d}I/\mathrm{d}V$ based on scattering formalism is given by:

$$G_{\mathrm{c}}(E) = \frac{e^2}{h} Tr\{I - r^{\dagger}_{\mathrm{ee}}(E)r_{\mathrm{ee}}(E) + r^{\dagger}_{\mathrm{he}}(E)r_{\mathrm{he}}(E)\}, \qquad (17)$$

Here, $r_{\mathrm{ee}}(r_{\mathrm{eh}})$ refers to the reflection coefficient for an incoming electron to be reflected as an electron (hole) at the artificially chosen interface (red dashed-line). Note that all the Andreev reflection processes at the physical interface (blue solid line) are essentially captured by r_{eh} at the artificial interface indicated by the red dashed-line (see schematic drawing in Supplementary Fig. 20).

With translational invariance in the *x*-direction, the total scattering matrix can be brought into sub-blocks characterized by momentum k_{x}. The reflection coefficients for a fixed k_{x} and energy E are given by the Lee-Fisher formula[37]

$$r_{\alpha\beta}(E,k_{\mathrm{x}}) = -I\sigma_0\delta_{\alpha\beta} + i\Gamma^{1/2}_{\alpha}(E,k_{\mathrm{x}})G^{\mathrm{R}}_{\mathrm{ii}}(E,k_{\mathrm{x}})\Gamma^{1/2}_{\beta}(E,k_{\mathrm{x}}), \qquad (18)$$

Here, $\alpha,\beta \in \{e,h\}$ are the electron/hole indices, σ_0 is the identity matrix in the spin space, and I is the identity matrix in the rest of the Hilbert space. $G^{\mathrm{R}}_{ii}(E,k_{\mathrm{x}}) = [E + i\eta - H_{\mathrm{tot}}(k_{\mathrm{x}})]^{-1}_{ii}$ is the retarded Green's function at the interface (red dashed line). Γ is the broadening function defined as $\Gamma(E,k_{\mathrm{x}}) = i[\Sigma(E,k_{\mathrm{x}}) - \Sigma^{\dagger}(E,k_{\mathrm{x}})]$, where $\Sigma(E,k_{\mathrm{x}})$ is the self-energy of the semi-infinite Cd_3As_2. The total differential conductance is thus obtained by summing over all k_x in the neighborhood of the Fermi surface:

$$G_{\mathrm{c}}(E) = \frac{e^2}{h}\sum_{k_{\mathrm{x}}} Tr\{I\sigma_0 - r^{\dagger}_{\mathrm{ee}}(k_{\mathrm{x}})r_{\mathrm{ee}}(k_{\mathrm{x}}) + r^{\dagger}_{\mathrm{he}}(k_{\mathrm{x}})r_{\mathrm{he}}(k_{\mathrm{x}})\}, \qquad (19)$$

Since transport experiments are carried out under finite temperature conditions (lowest T=2 K), we also incorporate the thermal effects on the conductance using the standard formula[38]

$$G_{\mathrm{c}}(E,T) = \frac{e^2}{h}\int dE'\, Tr\{I - r^{\dagger}_{\mathrm{ee}}(E')r_{\mathrm{ee}}(E') + r^{\dagger}_{\mathrm{he}}(E')r_{\mathrm{he}}(E')\}[-\frac{\partial f_{\mathrm{FD}}(E',T)}{\partial E'}], \quad (20)$$

Here, $f_{\mathrm{FD}}(E,T) = 1/[e^{\frac{E}{k_{\mathrm{B}}T}} + 1]$ is the Fermi-Dirac distribution function.

In the main text, we pointed out that a flat conductance plateau cannot be found when Fermi arc states are absent. Here, we demonstrate this result by artificially closing both the top and the bottom surfaces of Cd_3As_2 in our simulations, and calculate the $\mathrm{d}I/\mathrm{d}V$ for $E_{\mathrm{F}} = 70\ \mathrm{meV}$ with the same parameters used in Fig.3c of the main text. With the boundaries of the Dirac semimetal being closed, there exist no boundaries at the top/bottom surfaces to host Fermi arcs at the interface. As a result, the Andreev reflections in this case are solely driven by bulk state channels. As shown in Supplementary Fig. 21, in this case, a ZBBP stands in the midst of two separated coherence peaks, and the conductance plateau is absent. We note that, while this scenario without boundaries at top/bottom surfaces cannot be realized in real experimental settings, the theoretical result shown in Supplementary Fig. 21 identifies Fermi-arc states as the origin of the conductance plateau.

Supplementary Note 12. Josephson junctions in long junction limit

The $I_{\mathrm{c}}R_{\mathrm{n}}$ product is a characteristic junction parameter that provides useful information about superconducting transport through the Josephson junction. The $I_{\mathrm{c}}R_{\mathrm{n}}$ product is usually around Δ/e for short junctions, while it is much smaller for long junctions[39]. For our junction, we obtain $I_{\mathrm{c}}R_{\mathrm{n}}{\sim}17\ \mu\mathrm{V}$ at the lowest temperature, which is about 9 times smaller than $\Delta/e{\sim}150\ \mu\mathrm{V}$. This indicates that the junction is in the long junction limit, where the superconducting coherence length ξ_{N} is smaller than width between two Nb electrodes. The ξ_{N} can be evaluated by $\xi_{\mathrm{N}} = \frac{\hbar v_{\mathrm{F}}}{\pi\Delta}$ in the system. The Fermi velocity of thin Cd_3As_2 is $2.0 - 2.8 \times 10^5\ \mathrm{m/s}$ from Supplementary Table 1. Then, we get $\xi_{\mathrm{N}}{\sim}0.3 - 0.4\ \mu\mathrm{m}$ which is smaller than the effective width $L_{\mathrm{eff}}{\sim}1.4\ \mu\mathrm{m}$ estimated from the Fraunhofer pattern in SQI. In the diffusive junctions, the $I_{\mathrm{c}}R_{\mathrm{n}}$ product yields $I_{\mathrm{c}}R_{\mathrm{n}} \propto 1/W$ at low temperature which can explain the low $I_{\mathrm{c}}R_{\mathrm{n}}$ product observed.

Supplementary Note 13. Analysis of current density profile in Cd_3As_2 Josephson junctions

In a Josephson junction immersed in a perpendicular magnetic field B, the magnitude of the maximum critical current $I_{\mathrm{c}}^{\max}(B)$ depends strongly on the supercurrent density between the leads. Here we convert our measured interference patterns to their originating supercurrent density profiles. Our method follows the approach developed by Dynes and Fulton.[40]

At a fixed magnetic field, the total critical current through the Josephson junction is a phase-sensitive summation of supercurrent over the width of the junction. Suppose the supercurrent density profile $J_{\mathrm{S}}(z)$, its complex Fourier transform yields a complex critical current function $J_{\mathrm{C}}(\beta)$

$$J_{\mathrm{C}}(\beta) = \int_{-\infty}^{\infty} J_{\mathrm{S}}(x)\, e^{i\beta x} \mathrm{d}x, \tag{21}$$

where the normalized magnetic field unit $\beta = \frac{2\pi L_{\mathrm{eff}} B}{\Phi_0}$, and the magnetic flux quantum $\Phi_0 = h/2e$. The experimentally observed $I_{\mathrm{c}}^{\max}(B)$ is the magnitude of this

summation: $I_{\mathrm{C}}^{\max}(B)=|J_{\mathrm{C}}(\beta)|$. We use the even $(I_{\mathrm{E}}(\beta))$ and odd part $(I_{\mathrm{O}}(\beta))$ extracted from the $I_{\mathrm{C}}^{\max}(B)$. Then the $J_{\mathrm{C}}(\beta)$ can be expressed as

$$J_{\mathrm{C}}(\beta)=I_{\mathrm{E}}(\beta)+iI_{\mathrm{O}}(\beta), \tag{22}$$

The observed critical current $I_{\mathrm{C}}^{\max}(B)=\sqrt{I_{\mathrm{E}}^2(\beta)+I_{\mathrm{O}}^2(\beta)}$ is therefore dominated by $I_{\mathrm{E}}(\beta)$ except at its minima points. Approximately, $I_{\mathrm{E}}(\beta)$ is obtained by multiplying $I_{\mathrm{C}}^{\max}(\beta)$ with a flipping function that switches sign between adjacent lobes of the envelope function (Supplementary Fig. 22a-b). When $I_{\mathrm{E}}(\beta)$ is minimal, the odd part $I_{\mathrm{O}}(\beta)$ dominates the critical current. $I_{\mathrm{O}}(\beta)$ can then be approximated by interpolating between the minima of $I_{\mathrm{C}}^{\max}(\beta)$, and flipping sign between lobes (Supplementary Fig. 22c). A Fourier transform of the resulting complex $J_{\mathrm{C}}(\beta)$, over the sampling range b of β, yields the current density profile (Supplementary Fig. 22d):

$$J_{\mathrm{S}}(z)=\left|\frac{1}{2\pi}\int_{-\mathrm{b}/2}^{\mathrm{b}/2}J_{\mathrm{C}}(\beta)e^{-i\beta z}\mathrm{d}\beta\right|, \tag{23}$$

In Supplementary Fig. 23, we show the magnetic field dependence of the critical current. For the out-of-plane (B_{y}) direction, the critical current modulation resembles the Fraunhofer pattern, in which the critical current amplitude oscillates as a sine function with *B*. For a magnetic field B_{z}, the modulation is qualitatively different.

Supplementary Note 14. Control experiments for thick Cd_3As_2 Josephson junctions

Despite the successful demonstration of thin Cd_3As_2 Josephson junction dominated by surface as shown in the main text of device #06, we purposely use a thick Cd_3As_2 Josephson junction as control experiments. As shown in Supplementary Fig. 24a, the junction has $12\ \mu\mathrm{m}$ width and $400\ \mathrm{nm}$ length channel which is similar to the device #06. We deposited 200 nm Nb on top. Such a thick Cd_3As_2 (clear boundary at the sample region) is dominated by bulk states as demonstrated before. The temperature-dependent resistances of R_{12} and R_{23} are displayed in Supplementary Fig. 24b-c, respectively. Neither zero resistance nor Josephson effect is realized.

Supplementary Fig. 25b shows a plot of d*I*/d*V* as a function of the *V* measured by a four-probe method. A family of peak features is observed in both R_{12} and R_{23} channels, symmetrically around $V=0$. The conductivity at $V=0$ is finite which is different from the Josephson effect in device #06. The peaks correspond to the sub-harmonic energy-gap structure caused by multiple Andreev reflections (MARs)[41], with peak positions given by $eV_{\mathrm{n}}=2\Delta/n$ $(n=1,2,3,\cdots)$. MARs allow for Andreev channels to open up in the S-N-S junction at bias voltages below the superconducting energy gap 2Δ. These Andreev channels arise from a progressive increase of the incident carrier energy as the carrier reflects between the two interfaces. From a fit of the MARs peak positions in Supplementary Fig. 25c, we can determine that $2\Delta{\sim}2.2\ \mathrm{meV}$ corresponds to the Nb superconducting gap and the observed peaks correspond to $n=3,4,5,6,8,10,11,13,19,23,\cdots$ and $n=1,2,3,4,6,9,13,19,\cdots$ for R_{12} and R_{23}, respectively. we note that the position of the $n=1$ of R_{23} at high bias does not agree with the energy-gap value, likely due to the heating of the junction at high bias voltages[42] that would reduce Δ. These observed sub-harmonic peaks are

well reproducible and independent of sweep direction as shown in Supplementary Fig. 26a-b by changing the temperature and magnetic field, which also further proves the peak features related to the superconducting behavior. We did not observe other peak features due to our measurement resolution as well as the limits of thermal broadening. Besides, the first and second peaks of R_{12} are not seen in our measurement (voltage) limit. By applying 0.1 T magnetic field as displayed in Supplementary Fig. 26c, we are able to observe the differential resistance valley (or conductance peak) of the $n = 1, 2$ at high bias. We also observed MARs in another thick Cd_3As_2 Josephson junction as shown in Supplementary Fig. 27a-b. Such a clear signature of MARs has been reported in Ge/Si Josephson junctions before[42], indicating quite transparent contacts. The $n = 19$ subharmonic peaks shown in both R_{12} and R_{23} require that the charge carriers traverse the channel 19 times without being back-scattered inside the channel.

However, we are not able to observe Josephson effect in such a transparent junction while the junction resistance decreases to zero in thin Cd_3As_2 one with the same structure. It is hard to believe that this is caused by the fabrication process. If such MARs are dominated by bulk states, it is contradictory to the low transparency observed in Nb/thick Cd_3As_2 junctions as analyzed before. On the contrary, it is easier to believe that the MARs are dominated by the surface states which couple well with the superconducting Nb. The low density of surface states with low transparency of bulk states in thick Cd_3As_2 makes it difficult to achieve Josephson effect which usually happens in graphene Josephson junction.

Supplementary Note 15. Other possible reasons for surface supercurrent

Since it is quite challenging to rule out other possibilities in our transport measurements, we discuss three effects which may destroy the supercurrent uniformity.

First, surface doping by charged impurities may result in the non-uniform supercurrent. We point out that for this alternative mechanism to be compatible with our observed interference pattern, one would require that the charge impurities have a rather non-uniform distribution on different surface terminations. In particular, if the SQUID-like pattern is induced by impurities, these impurities should be predominantly concentrated on the top and bottom surfaces of the junction, while not on the side surfaces, *i.e.*, [001] surfaces with their surface normal vectors parallel to the principal *z*-axis. However, as we explain below, this is unlikely to occur in our realistic experimental set-up.

In our experiment, impurities are most likely to be induced by two processes: (a) Nb deposition and (b) Cd_3As_2 nanoplate growth.

(a) For the Nb deposition, the impurities are likely to be induced only at the interface of Nb/Cd_3As_2 region as shown in the blue region in Supplementary Fig. 28. It is hard to induce impurities on the whole top and bottom surfaces. Besides, we used HF to etch and clean the surface. The sample was put into HF for 5 seconds right before sputtering. Therefore, the impurities on the top layer, if any, should be reduced largely.

(b) During the Cd_3As_2 nanoplate growth process, the charge impurities can dope impurities states at both surfaces. However, this cannot change the density of the bulk states. Our result shows the supercurrent density in bulk is almost at a similar level with the background in Fig. 5f. Impurities alone do not have this strong effect to prevent the

bulk supercurrent density. Furthermore, these surface impurities' contribution should be reduced in thicker Cd_3As_2. However, no Josephson effect occurs in thick devices as shown in Supplementary Fig. 23-24, contradictory to the hypothesis.

Secondly, the band-bending effect is possible which is previously observed in graphene Josephson junction[43] with edge supercurrent. The atomically sharp edges provide an edge potential along y axis and a natural vehicle for band bending near the boundary, which then confines the electronic waves in the direction transverse to the edge[44, 45]. The resulting guided fibre-optic modes are situated outside the Dirac continuum. These guided modes can result in edge superconductivity with non-uniform supercurrent density. Thus, the SQUID pattern can be obtained in superconducting quantum interference (SQI) measurement by applying out-of-plane magnetic field along z-axis.

However, in our Cd_3As_2 Josephson junction, we do not observe the edge supercurrent in Fig. 5c-d indicating no band bending effect along the in-plane direction. However, the supercurrent density is higher at the top and bottom surface. It means that the band bending can only take place on the surfaces. Therefore, if there is a band bending effect, this band bending effect in Cd_3As_2 is highly anisotropic which is unusual.

At last, the surface supercurrent may also relate to finite size effect. In a superconductor/metal hybrid structure, the superconducting proximity effect can transit the normal metal layer into fully superconducting state when the thickness of normal metal is thinner than the coherence length of the superconductor ($t < \xi_S$). The surface is easier to induce proximity effect in this case. This effect is widely used in TI/SC junction to induce superconductivity into TI[46]. The length, width and height of the superconducting junction channel are $L = 500$ nm, $W = 7$ μm and $t = 120$ nm, respectively. The coherence length of Nb[47] is $\xi_S = 38$ nm, which is much smaller to the length scales in all dimensions of our Cd_3As_2 devices. This indicates a low possibility of finite size effect. Moreover, even if small finite size effects occurs, the bulk region should exhibit finite supercurrent density rather than being ultralow level as we observed.

In summary, the three possibilities cannot be completely ruled out. However, based on the analysis above, the assumptions behind these competing interpretations either cannot be met by our realistic experimental conditions, or require rather unusual anisotropic configurations. In contrast, the intrinsic physical properties of surface Fermi arcs, such as their anisotropic surface distribution and their thickness-independent behaviors, naturally provide consistent signatures with our experiment. The nature of the superconducting proximity effect in Cd_3As_2 Josephson junction is coherent Cooper pairs spreading out layer by layer. Without the loop formed by Fermi arcs, the top and bottom surfaces in our Cd_3As_2 are almost disconnected due to the very low density of states in the bulk, as seen by the extremely low supercurrent distribution in the bulk region in Fig. 5f. Thus, the localized supercurrent density on the surfaces observed in our experiment can hardly be created without Fermi arcs. Therefore, we believe that the explanation by Fermi arc is more reasonable than these three effects.

Moreover, two pieces of evidence suggest the non-trivial surface states responsible

for the surface superconductivity: firstly, the SQUID pattern observed in Josephson junctions suggests that the surface states form a closed loop which covers both top and bottom surfaces because only the top surface is directly coupled to superconducting Nb electrodes. Thus, without Fermi arc states that connect the top and bottom surfaces, the induced surface pairing can hardly be strong and stable on both surfaces. This robust loop is rather unlikely due to other effects that can be easily scattered into the bulk. Secondly, our BICP signal in Nb/Cd_3As_2 interface is generally observed in different thin Cd_3As_2 samples, which indicates that the surface state contribution is not very sensitive to details of the system such as thickness (device #1-4 not for device #5), Fermi energies or disorder configurations. Therefore, we believe that the explanation by Fermi arcs is more reasonable than these competing interpretations.

Supplementary References

1. Zhang, C., *et al.* Evolution of Weyl orbit and quantum Hall effect in Dirac semimetal Cd3As2. *Nat. Commun.* **8**, 1272 (2017).
2. Tinkham, M. *Introduction to superconductivity*. McGraw-Hill (1975).
3. Huang, C., *et al.* Inducing Strong Superconductivity in WTe2 by a Proximity Effect. *ACS Nano* **12**, 7185-7196 (2018).
4. Kastalsky, A., *et al.* Observation of pair currents in superconductor-semiconductor contacts. *Phys. Rev. Lett.* **67**, 3026-3029 (1991).
5. Kashiwaya, S., *et al.* Theory for Tunneling Spectroscopy of Anisotropic Superconductors. *Phys. Rev. B* **53**, 2667-2676 (1996).
6. Dynes, R. C., Narayanamurti, V. & Garno, J. P. Direct Measurement of Quasiparticle-Lifetime Broadening in a Strong-Coupled Superconductor. *Phys. Rev. Lett.* **41**, 1509-1512 (1978).
7. Pleceník, A., *et al.* Finite-Quasiparticle-Lifetime Effects in the Differential Conductance of Bi2Sr2CaCu2Oy/Au Junctions. *Phys. Rev. B* **49**, 10016-10019 (1994).
8. Mühlschlegel, B. Die thermodynamischen Funktionen des Supraleiters. *Zeitschrift für Physik* **155**, 313-327 (1959).
9. Yabuki, N., *et al.* Supercurrent in Van Der Waals Josephson Junction. *Nat. Commun.* **7**, 10616 (2016).
10. Taboryski, R., *et al.* Andreev reflections at interfaces between δ-doped GaAs and superconducting Al films. *Appl. Phys. Lett.* **69**, 656-658 (1996).
11. Kutchinsky, J., *et al.* Decay Lengths for Diffusive Transport Activated by Andreev Reflections in Al/n-GaAs/Al Superconductor-Semiconductor-Superconductor Junctions. *Phys. Rev. Lett.* **78**, 931-934 (1997).
12. Yang, F., *et al.* Proximity effect at superconducting Sn-Bi2Se3 interface. *Phys. Rev. B* **85**, 104508 (2012).
13. Nguyen, C., Kroemer, H. & Hu, E. L. Anomalous Andreev conductance in InAs-AlSb quantum well structures with Nb electrodes. *Phys. Rev. Lett.* **69**, 2847-2850 (1992).
14. Xiong, P., Xiao, G. & Laibowitz, R. B. Subgap and above-gap differential resistance anomalies in superconductor-normal-metal microjunctions. *Phys. Rev. Lett.* **71**, 1907-1910 (1993).
15. van Wees, B. J., *et al.* Excess conductance of superconductor-semiconductor interfaces due to phase conjugation between electrons and holes. *Phys. Rev. Lett.* **69**, 510-513 (1992).
16. Xiong, P., Xiao, G. & Laibowitz, R. B. Subgap and above-gap differential resistance anomalies in superconductor-normal-metal microjunctions. *Phys. Rev. Lett.* **71**, 1907-1910 (1993).
17. Nadj-Perge, S., *et al.* Observation of Majorana fermions in ferromagnetic atomic chains on a superconductor. *Science* **346**, 602 (2014).
18. Xu, S., *et al.* Momentum-space imaging of Cooper pairing in a half-Dirac-gas topological superconductor. *Nat. Phys.* **10**, 943 (2014).
19. Nichele, F., *et al.* Scaling of Majorana Zero-Bias Conductance Peaks. *Phys. Rev. Lett.* **119**, 136803 (2017).
20. Beenakker, C. W. J. Quantum transport in semiconductor-superconductor microjunctions. *Phys. Rev. B* **46**, 12841-12844 (1992).
21. Kashiwaya, S., *et al.* Origin of zero-bias conductance peaks in high Tc superconductors. *Phys. Rev. B* **51**, 1350-1353 (1995).
22. Deutscher, G. Andreev-Saint-James Reflections: A Probe of Cuprate Superconductors. *Rev.*

Mod. Phys. **77**, 109-135 (2005).

23. Daghero, D. & Gonnelli, R. S. Probing Multiband Superconductivity by Point-Contact Spectroscopy. *Supercond. Sci. Technol.* **23**, 043001 (2010).
24. Laube, F., *et al.* Spin-Triplet Superconductivity in Sr2RuO4 Probed by Andreev Reflection. *Phys. Rev. Lett.* **84**, 1595-1598 (2000).
25. Buchholtz, L. J. & Zwicknagl, G. Identification of p-wave superconductors. *Phys. Rev. B* **23**, 5788-5796 (1981).
26. Di Bernardo, A., *et al.* p-wave triggered superconductivity in single-layer graphene on an electron-doped oxide superconductor. *Nat. Commun.* **8**, 14024 (2017).
27. Wang, H., *et al.* Observation of Superconductivity Induced by a Point Contact on 3D Dirac Semimetal Cd3As2 Crystals. *Nat. Mater.* **15**, 38-42 (2016).
28. Liu, Z. K., *et al.* A stable three-dimensional topological Dirac semimetal Cd3As2. *Nat. Mater.* **13**, 677 (2014).
29. Jeon, S., *et al.* Landau quantization and quasiparticle interference in the three-dimensional Dirac semimetal Cd3As2. *Nat. Mater.* **13**, 851 (2014).
30. Zhang, C., *et al.* Room-temperature chiral charge pumping in Dirac semimetals. *Nat. Commun.* **8**, 13741 (2017).
31. Li, C., *et al.* Giant negative magnetoresistance induced by the chiral anomaly in individual Cd3As2 nanowires. *Nat. Commun.* **6**, 10137 (2015).
32. Moll, P. J. W., *et al.* Transport evidence for Fermi-arc-mediated chirality transfer in the Dirac semimetal Cd3As2. *Nature* **535**, 266 (2016).
33. Zhao, Y., *et al.* Anisotropic Fermi Surface and Quantum Limit Transport in High Mobility Three-Dimensional Dirac Semimetal Cd3As2. *Phys. Rev. X* **5**, 031037 (2015).
34. Wang, C. M., Lu, H.-Z. & Shen, S.-Q. Anomalous Phase Shift of Quantum Oscillations in 3D Topological Semimetals. *Phys. Rev. Lett.* **117**, 077201 (2016).
35. Reifenberger, R. *Magnetic Oscillations in Metals*. Cambridge University Press (1984).
36. Liang, T., *et al.* Ultrahigh mobility and giant magnetoresistance in the Dirac semimetal Cd3As2. *Nat. Mater.* **14**, 280 (2014).
37. Fisher, D. S. & Lee, P. A. Relation between conductivity and transmission matrix. *Phys. Rev. B* **23**, 6851-6854 (1981).
38. Anantram, M. P. & Datta, S. Current fluctuations in mesoscopic systems with Andreev scattering. *Phys. Rev. B* **53**, 16390 (1996).
39. Dubos, P., *et al.* Josephson critical current in a long mesoscopic S-N-S junction. *Phys. Rev. B* **63**, 064502 (2001).
40. Dynes, R. C. & Fulton, T. A. Supercurrent Density Distribution in Josephson Junctions. *Phys. Rev. B* **3**, 3015-3023 (1971).
41. Octavio, M., *et al.* Subharmonic energy-gap structure in superconducting constrictions. *Phys. Rev. B* **27**, 6739-6746 (1983).
42. Xiang, J., *et al.* Ge/Si nanowire mesoscopic Josephson junctions. *Nat. Nanotechnol.* **1**, 208 (2006).
43. Allen, M. T., *et al.* Spatially resolved edge currents and guided-wave electronic states in graphene. *Nat. Phys.* **12**, 128 (2015).
44. Zhang, F., He, Y. & Chen, X. Guided modes in graphene waveguides. *Appl. Phys. Lett.* **94**, 212105 (2009).

45. Williams, J. R., *et al.* Gate-controlled guiding of electrons in graphene. *Nat. Nanotechnol.* **6**, 222 (2011).
46. Wang, M.-X., *et al.* The Coexistence of Superconductivity and Topological Order in the Bi2Se3 Thin Films. *Science* **336**, 52 (2012).
47. Kittel, C. *Introduction to solid state physics*. Wiley New York (1976).
